# Prevalence and correlates of manic/hypomanic and depressive predominant polarity in bipolar disorder: systematic review and meta-analysis

**DOI:** 10.1192/bjo.2024.51

**Published:** 2024-05-06

**Authors:** Francesco Bartoli, Carlo Bassetti, Marco Gazzola, Letizia Gianfelice, Daniele Cavaleri, Cristina Crocamo, Giuseppe Carrà

**Affiliations:** Department of Medicine and Surgery, University of Milano-Bicocca, Monza, Italy; Department of Medicine and Surgery, University of Milano-Bicocca, Monza, Italy; and Division of Psychiatry, University College London, UK

**Keywords:** Bipolar disorder, predominant polarity, meta-analysis, mania, bipolar depression

## Abstract

**Background:**

Identification of the predominant polarity, i.e. hypomanic/manic (mPP) or depressive predominant polarity (dPP), might help clinicians to improve personalised management of bipolar disorder.

**Aims:**

We performed a systematic review and meta-analysis to estimate prevalence and correlates of mPP and dPP in bipolar disorder.

**Method:**

The protocol was registered in the Open Science Framework Registries (https://doi.org/10.17605/OSF.IO/8S2HU). We searched main electronic databases up to December 2023 and performed random-effects meta-analyses of weighted prevalence of mPP and dPP. Odds ratios and weighted mean differences (WMDs) were used for relevant correlates.

**Results:**

We included 28 studies, providing information on rates and/or correlates of mPP and dPP. We estimated similar rates of mPP (weighted prevalence = 30.0%, 95% CI: 23.1 to 37.4%) and dPP (weighted prevalence = 28.5%, 95% CI: 23.7 to 33.7%) in bipolar disorder. Younger age (WMD = −3.19, 95% CI: −5.30 to −1.08 years), male gender (odds ratio = 1.39, 95% CI: 1.10 to 1.76), bipolar-I disorder (odds ratio = 4.82, 95% CI: 2.27 to 10.24), psychotic features (odds ratio = 1.56, 95% CI: 1.01 to 2.41), earlier onset (WMD = −1.57, 95% CI: −2.88 to −0.26 years) and manic onset (odds ratio = 13.54, 95% CI: 5.83 to 31.46) were associated with mPP (*P* < 0.05). Depressive onset (odds ratio = 12.09, 95% CI: 6.38 to 22.90), number of mood episodes (WMD = 0.99, 95% CI: 0.28 to 1.70 episodes), history of suicide attempts (odds ratio = 2.09, 95% CI: 1.49 to 2.93) and being in a relationship (odds ratio = 1.98, 95% CI: 1.22 to 3.22) were associated with dPP (*P* < 0.05). No differences were estimated for other variables.

**Conclusions:**

Despite some limitations, our findings support the hypothesis that predominant polarity might be a useful specifier of bipolar disorder. Evidence quality was mixed, considering effects magnitude, consistency, precision and publication bias. Different predominant polarities may identify subgroups of patients with specific clinical characteristics.

Bipolar disorder is a severe and chronic condition, affecting about 1–2% of the general population.^1,2^ Its clinical course is characterised by mood recurrencies, in which depressive episodes alternate with manic or hypomanic episodes, according to the conventional differentiation between bipolar-I disorder (BD-I) and bipolar-II disorder (BD-II).^3^ However, despite epidemiological assumptions that people with bipolar disorder spend more time affected by depression than by mania,^4,5^ the clinical course and trajectories of bipolar disorder may be rather heterogeneous.^6,7^ In particular, it has been proposed that a more fine-grained classification of bipolar disorder should consider whether the clinical course is characterised by a depressive (dPP) or manic/hypomanic (mPP) predominant polarity.^8,9^ The concept of predominant polarity was first introduced by Jules Angst,^10^ based on a study investigating 95 individuals with bipolar disorder. Participants were subdivided into three subtypes according to their mood recurrencies, i.e. the preponderantly manic, the preponderantly depressed and the nuclear type, in which there was a balanced proportion of depressive and manic episodes.^10^ Thereafter, Colom et al^11^ provided a more detailed definition of predominant polarity in bipolar disorder, proposing that to define mPP, manic/hypomanic episodes should represent at least two-thirds of the overall number of lifetime mood episodes. On the other hand, dPP requires that among lifetime mood episodes, at least two-thirds are depressive. Finally, an undetermined predominant polarity should be considered if there is a sufficiently balanced proportion between manic and depressive episodes in the clinical course of bipolar disorder, without any clear mood episode predominance.^11^ Alternative definitions have been proposed,^12^ together suggesting that the identification of the predominant polarity might help clinicians to improve the personalised management of bipolar disorder by making its clinical trajectories clearer.^9^ Indeed, available evidence suggests that mPP or dPP may influence individual response to acute and long-term treatment for bipolar disorder, as well as the effectiveness of psychopharmacological agents used for the stabilisation phase.^8,13^ Exploring the hypothesis of predominant polarity as a possible clinical specifier, previous reviews have suggested that mPP and dPP might involve approximately half of all people with bipolar disorder and might be associated with particular individual characteristics.^8,14^ However, despite the growing scientific interest in this field,^15–18^ no systematic analyses on rates and individual characteristics associated with mPP versus dPP are available so far. To shed light on this topic, we performed a systematic review and meta-analysis of observational studies aimed at identifying the prevalence and clinical correlates of different mood predominance types in bipolar disorder, as well as assessing the quality of evidence in terms of strength, precision, consistency and risk of publication bias.

## Method

### Study design and protocol

This systematic review and meta-analysis is reported following the Meta-analysis Of Observational Studies in Epidemiology guidelines.^19^ The study protocol was registered on 27 November 2023 in Open Science Framework Registries (https://doi.org/10.17605/OSF.IO/8S2HU) and amended on 8 December 2023 because of changes in the search strategy.

### Eligibility criteria

We included any observational studies (a) providing information on prevalence rates of mPP and dPP in people with bipolar disorder and (b) comparing them with respect to one or more sociodemographic or clinical characteristics. To be considered, studies had to include at least ten individuals in each group (mPP and dPP). Moreover, in order to improve consistency across studies and to reduce the risk of misclassification bias, we included only studies which used the recommended Colom's definition for predominant polarity.^11^ Based on this, mPP and dPP are defined as a lifetime ratio ≥2:1 of either hypomanic/manic episodes or depressive episodes, respectively. This restrictive definition, splitting patients in three categories (mPP, dPP and undetermined predominant polarity), is considered to be more stable and conservative over time than other definitions,^12^ making patients less likely to be switched from one category to another across different episodes.^9^ We excluded studies (a) not providing information on predominant polarity, (b) not comparing mPP and dPP in terms of relevant sociodemographic or clinical characteristics, (c) including samples with a mean age <18 years, (d) using definitions of predominant polarity based on different criteria, and (e) published before the release date of DSM-IV.^20^ In order to avoid duplicate results, we excluded data on correlates derived from the same sample, including only the study that provided the larger amount of information. Finally, we excluded scientific reports not undergoing a peer-review process, such as conference abstracts, dissertations and grey literature.

### Article screening

We searched the Embase, PubMed, APA PsycInfo (via ProQuest), and Emcare (via Ovid) databases for articles indexed up to 8 December 2023, without any language restrictions. We used the following search phrases adapted for each database: (a) Embase: ‘bipolar disorder’:ti,ab,kw AND ‘predominant polarity’:ti,ab,kw; (b) PubMed: bipolar [Title/Abstract] AND predominant polarity [Title/Abstract]; (c) PsycInfo: tiab(bipolar disorder) AND tiab(predominant polarity); (d) Ovid Emcare: (bipolar and (predominance or ‘predominant polarity’)).ti. or (bipolar and (predominance or ‘predominant polarity’)).ab. An additional manual search of studies included in two relevant reviews^8,14^ was carried out to check for further potentially eligible studies. References were managed using EndNote web software. After the preliminary screening based on titles and abstracts had been completed, full texts were retrieved to assess the final eligibility of studies. These procedures were completed by three authors (C.B., M.G. and L.G.) independently, and reasons for exclusion after full-text review were recorded. Disagreements concerning suitability for inclusion were resolved by discussion and consensus involving all authors.

### Data extraction

Data were extracted between 11 and 13 December 2023, using a standard template to collect key information from all eligible studies: year of publication; country; setting; inclusion and exclusion criteria; sample size, mean age, and sex proportion; methods used to define predominant polarity; prevalence rates of mPP and dPP; and sociodemographic and clinical correlates of mPP versus dPP. Four authors (F.B., C.B., M.G. and L.G.) independently extracted data and blindly cross-checked them for accuracy.

### Data analysis

Meta-analyses of mPP and dPP prevalence in bipolar disorder were based on random-effects weighted proportions with 95% confidence intervals using arcsine-based transformation. Considering the expected low consistency of meta-analyses of prevalence rates,^21^ subgroup analyses were run to test potential variations in mPP and dPP prevalence rates by the geographical area of included studies. An omnibus test from the random-effects meta-regression was performed to test the overall moderating effects of subgroups. Moreover, in order to deal with a skewed distribution of prevalence rates, we reported the overall median and interquartile range of the mPP and dPP point prevalences for descriptive purposes. Weighted differences in arcsine-transformed proportions (WPDs) were estimated for both overall and subgroup analyses by geographical area.

To compare mPP and dPP for relevant correlates, random-effects meta-analyses were conducted for variables with data available from at least five different studies. *P* < 0.05 was used as the threshold for statistical significance. We used odds ratios and weighted mean differences (WMDs) with 95% confidence intervals for categorical and continuous variables, respectively. Heterogeneity across studies was evaluated according to standard cut-offs for *I*2 statistics to measure inconsistency of meta-analyses on correlates.^22^ Publication bias was assessed using Egger's test for meta-analyses with data available from at least ten studies.^23^ To evaluating the magnitude and precision of the effects (see ‘Grading of the evidence’ section), each WMD was converted into the equivalent effect size (standardised mean difference [SMD]), performing relevant meta-analysis, while each odds ratio was converted into an SMD by dividing the relevant ln(OR) by 1.81.^24^ Conventional cut-offs (0.2 small, 0.5 medium, 0.8 large) were used to interpret the magnitude of the effect.^25^ Data analyses were performed using Stata statistical software, release 17 (StataCorp LLC, 2021). OpenMeta[Analyst] software^26^ was used to generate forest plots.

### Grading of the evidence

Following a similar approach used in recent meta-analyses,^27,28^ we used GRADE (Grading of Recommendations, Assessment, Development, and Evaluations) items,^29^ adapted for non-interventional observational studies, to classify the quality of evidence as high, moderate, low or very low for each variable showing a statistically significant estimate (*P* < 0.05).

First, we assessed the consistency of findings according to the *I*^2^ value. We downgraded by one level the quality of evidence if inconsistency was estimated (*I*^2^ ≥ 50%).

Second, we evaluated the precision of findings by checking the width of the equivalent effect size 95% CI. We downgraded the quality of evidence by one level if (a) the 95% CI width of the equivalent effect size was ≥0.40 for meta-analyses showing small or medium effect sizes, or (b) the 95% lower and upper confidence limits of the equivalent effect size (SMD) were not both ≥0.80 for meta-analyses showing large effect sizes.

In addition, we assessed the risk of publication bias, downgrading the quality of evidence by one level if (a) meta-analyses included fewer than ten studies, or (b) the Egger's test *P*-value was <0.10 for meta-analyses including at least ten studies.

Finally, we evaluated the magnitude of the effect, upgrading the quality of evidence by one level if the magnitude of the equivalent effect size was large (SMD ≥ 0.80).

## Results

### Study selection

**Fig. 1:**
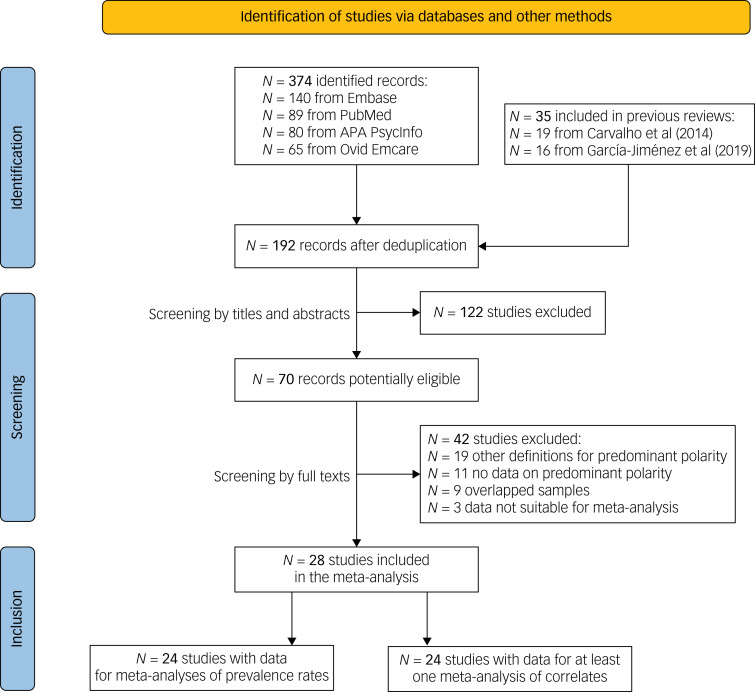
Flow diagram of included and excluded studies.

The systematic search on relevant databases generated 374 records, namely 140 from Embase, 89 from PubMed, 80 from APA PsycInfo and 65 from Ovid Emcare. After deduplication, there were 192 articles left to be screened, including additional studies retrieved from the reference lists of the two reviews.^8,14^ After screening by titles and abstracts, 70 studies were identified as potentially eligible. Following the final screening based on full texts, 28 studies met the eligibility criteria and were included in the meta-analysis.^12,15–18,30–52^ A flowchart with details of screening, the study selection process and reasons for exclusion is presented in Fig. 1.

### Study characteristics

Studies were published between 2009^40,50^ and 2023.^31^ All studies, with the exception of one reported in Spanish,^43^ were written in English. The sample sizes varied between 42^31^ and 788.^50^ The majority of studies (*k* = 16) were conducted in Europe, i.e. six were from Italy,^30,38–41,45^ three from Spain,^15,46,49^ two from France^32,42^ and Germany,^51,52^ and one each from Belgium,^18^ Finland^17^ and Greece.^31^ Five studies were conducted in Asia: four in India^16,36,44,47^ and one in Singapore;^37^ and five studies in South America, i.e. three were from Brazil^33,34,48^ and two from Colombia.^35,43^ Two studies were based on data from multiple countries of different geographical areas.^12,50^ Study characteristics are reported in Table 1. Some studies were likely to have a partial overlap between included samples, i.e. (a) Belizario et al (2019)^33^ and Belizario et al (2018);^34^ (b) Fico et al (2022)^15^ and Popovic et al (2014);^46^ and (c) Pacchiarotti et al (2011)^45^ and Mazzarini et al (2009).^40^ We prioritised data from Belizario et al (2019),^33^ Fico et al (2022)^15^ and Pacchiarotti et al (2011),^45^ respectively, as these studies were all based on larger sample sizes. For meta-analyses, we used data from smaller studies^34,40,46^ only if these were not provided by the main study.

**Table 1:** Characteristics of included studies

Study	Country	Setting	Sample size (*N)*	BD-I (%)	Age, mean or median	Males (%)	Age at onset in years, mean or median	Predominant polarity assessment
Albert et al (2021)^30^	Italy	In-patient and out-patient	653	40.1	50.6	40.6	30.3	Interview and clinical charts
Argyropoulos et al (2023)^31^	Greece	Out-patient	42	71.4	46.6	47.6	NR	Interview (patient and caregiver) and clinical records
Azorin et al (2015)^32^	France	In-patient and out-patient	278	NR	NR	NR	NR	Interview and clinical records
Baldessarini et al (2012)^12^	Multiple countries	NR	928	100	36.5	48.4	22.0	Interview
Belizario et al (2018)^33^	Brazil	Out-patient	87	85.1	49.2	26.4	23.2	Interview, clinical records and charts
Belizario et al (2019)^34^	Brazil	Out-patient	248	NR	NR	NR	NR	Interview
Carreño Ruiz et al (2022)^35^	Colombia	NR	77	100	43	63.6	20	Interview
Fico et al (2022)^15^	Spain	In-patient and out-patient	708	71	45.3	45.1	27.3	Interview (patient and caregiver)
Ghosal et al (2021)^36^	India	NR	100	NR	35.8	61.0	NR	Interview
Grover et al (2021)^16^	India	Out-patient	773	92.4	45.7	63.6	26.3	Interview
Gunasekaran et al (2023)^37^	Singapore	Out-patient	74	90.5	38.3	45.9	26.1	Interview
Janiri et al (2017)^38^	Italy	In-patient and out-patient	218	38.5	45.0	47.7	28.4	Interview
Janiri et al (2020)^39^	Italy	Out-patient	172	58.7	43.6	47.7	NR	Interview
Mazzarini et al (2009)^40^	Italy	In-patient	124	NR	NR	NR	NR	Interview
Murru et al (2015)^41^	Italy	Out-patient	119	67.2	52.7	44.5	28.5	Clinical records
Murru et al (2018)^42^	France	Out-patient	468	57.3	47.7	41.0	NR	Interview
Obando et al (2012)^43^	Colombia	NR	94	100	50	NR	24.7	Interview
Pacchiarotti et al (2011)^44^	Italy	In-patient	134	NR	NR	56.0	25.9	Interview and clinical records
Pal et al (2020)^45^	India	Out-patient	75	NR	32.6	72	23.5	Interview
Pallaskorpi et al (2019)^17^	Finland	In-patient and out-patient	188	46.8	37.6	46.8	NR	Interview
Popovic et al (2014)^46^	Spain	NR	604	NR	NR	NR	NR	Interview (patient and caregiver)
Rangappa et al (2016)^47^	India	In-patient	285	100	33.6	56.8	22.1	Interview (patient and caregiver) and clinical records
Rosa et al (2008)^48^	Brazil	Out-patient	149	NR	NR	NR	NR	Interview (patient and caregiver)
Sentissi et al (2019)^18^	Belgium	In-patient and out-patient	356	58.9	47.5	42.1	NR	Interview
Vidal-Rubio et al (2018)^49^	Spain	Out-patient	118	66.1	52.3	39.0	NR	Interview
Vieta et al (2009)^50^	Multiple countries	In-patient and out-patient	788	NR	NR	NR	NR	Interview
Volkert et al (2014)^51^	Germany	In-patient and out-patient	336	NR	NR	NR	NR	Interview (patient and caregiver) and clinical records
Wenzel et al (2022)^52^	Germany	In-patient and out-patient	52	53.4	45.8	50.0	28.3	Interview (patient and caregiver) and clinical charts

BD-I, bipolar-I disorder; NR, not reported.

### Prevalence of hypomanic/manic and depressive predominant polarity in bipolar disorder

Twenty-four studies^12,15–18,30–32,34–39,41–45,47–51^ including 7381 individuals with bipolar disorder were included in the meta-analyses on weighted prevalence of mPP and dPP. We found similar rates of mPP (weighted prevalence = 30.0%, 95% CI: 23.1 to 37.4%; Supplementary Fig. 1 available at https://doi.org/10.1192/bjo.2024.51) and dPP (weighted prevalence = 28.5%, 95% CI: 23.7 to 33.7%; Supplementary Fig. 2) across studies (WPD = 1.6%, 95% CI: −9.6 to 12.9%, *P* = 0.78; Supplementary Fig. 3). The risk of publication bias was low (Egger's coefficient: 1.60, *P* = 0.63). However, statistically significant variations were estimated by subgroup analyses (omnibus *P* < 0.001). In Europe (*k* = 13; *N* = 3790), rates of mPP (weighted prevalence = 20.1%, 95% CI: 15.2 to 25.4%) were significantly lower (WPD = −15.0%, 95% CI: −24.3 to −5.7%; *p* = 0.002) than rates of dPP (weighted prevalence = 33.2%, 95% CI: 25.3 to 41.6%). The opposite trend was estimated for studies conducted in Asia (*k* = 5; *N* = 1307): mPP affected more than half of people with bipolar disorder (weighted prevalence = 52.7%, 95% CI: 35.8 to 69.2%), whereas dPP occurred in around a fifth of patients (weighted prevalence = 20.5, 95% CI: 15.6 to 26.0%). The difference was statistically significant (WPD = 33.3%, 95% CI: 11.1 to 55.5%; *P* = 0.003). A similar trend was observed in studies carried out in South America, although the difference between mPP (weighted prevalence = 42.7%, 95% CI: 28.3 to 57.8%) and dPP (weighted prevalence = 23.7%, 95% CI: 15.1 to 33.4%) was not statistically significant (WPD = 20.8%, 95% CI: −4.8 to 46.3%), possibly because of the limited number of included studies (*k* = 4) and the small resulting sample size (*N* = 568). Results are reported in Table 2.

**Table 2:** Meta-analyses of prevalence rates of hypomanic/manic and depressive predominant polarity in people with bipolar disorder by geographical area

Group		Overall	Europe	Asia	South America
	*k*	24	13	5	4
	*N*	7381	3790	1307	568
Hypomanic/manic predominant polarity	WP [95% CI]	30.0% [23.1 to 37.4%]	20.1% [15.2 to 25.4%]	52.7% [35.8 to 69.2%]	42.7% [28.3 to 57.8%]
	Median (IQR)	31.2% (16.0 to 42.4%)	18.6% (13.7 to 31.2%)	45.8% (44.0 to 49.3%)	41.1% (31.0 to 53.4%)
Depressive predominant polarity	WP [95% CI]	28.5% [23.7 to 33.7%]	33.2% [25.3 to 41.6%]	20.5% [15.6 to 26.0%]	23.7% [15.1 to 33.4%]
	Median (IQR)	28.8% (20.1 to 36.1%)	36.1% (20.1 to 39.9%)	20.6% (17.0 to 28.0%)	27.4% (21.2 to 30.2%)
Comparison between predominant polarities	WPD [95% CI]	1.6% [−9.6 to 12.9%]	−15.0% [−24.3 to −5.7%]	33.3% [11.1 to 55.5%]	20.8% [−4.8 to 46.3%]
	*P*-value	*P* = 0.78	*P* = 0.002	*P* = 0.003	*P* = 0.11

IQR, interquartile range, expressed as (1st quartile to 3rd quartile); *k*, number of included studies; *N*, number of study participants; WP, weighted prevalence; WPD, weighted prevalence difference.

### Selection of variables

We extracted data for 22 correlates from at least five studies based on unique samples. Twenty-four studies^12,15–18,30–36,38–40,43,44,46–52^ had data suitable for at least one correlate. We did not consider manic and depressive symptoms or the lifetime number of manic/hypomanic and depressive episodes, even if these variables were based on more than five studies, owing to the inherent association with the corresponding predominant polarity. Moreover, we did not consider pharmacological treatments because of the extreme variability across studies in terms of current and lifetime treatments. We distinguished variables included for meta-analyses as ‘variables associated with mPP’, ‘variables associated with dPP’ and ‘variables not associated with any predominant polarity’.

### Variables associated with hypomanic/manic predominant polarity

A summary of findings and related details on quality of evidence are reported in Tables 3 and 4, respectively.

**Table 3:** Factors associated with hypomanic/manic or depressive predominant polarity: summary of findings

Variable	*k*	*N*	Effect size [95% CI]	Equivalent effect size [95% CI]	*P*-value	*I*^2^	Egger's test coeff. (*P*-value)
Factors associated with hypomanic/manic predominant polarity (versus depressive predominant polarity)							
Age (years)	18	2827	WMD = −3.19 years [−5.30 to −1.08 years]	SMD = −0.26 [−0.43 to −0.08]	0.003	74.3%	coeff.: −0.99 (*P* = 0.43)
Male gender	19	3335	OR = 1.39 [1.10 to 1.76]	SMD = 0.18 [0.05 to 0.31]	0.005	45.9%	coeff.: −0.28 (*P* = 0.73)
Age at onset (years)	13	2494	WMD = −1.57 years [−2.88 to −0.26 years]	SMD = −0.15 [−0.27 to −0.02]	0.019	42.3%	coeff.: 0.44 (*P* = 0.69)
Manic polarity of first episode	8	1557	OR = 13.54 [5.83 to 31.46]	SMD = 1.44 [0.97 to 1.90]	0.001	84.8%	N/A
Bipolar-I disorder	12	1661	OR = 4.82 [2.27 to 10.24]	SMD = 0.87 [0.45 to 1.28]	0.001	81.8%	coeff.: 1.08 (*P* = 0.54)
Psychotic features	10	1970	OR = 1.56 [1.01 to 2.41]	SMD = 0.25 [0.01 to 0.49]	0.047	66.1%	coeff.: −0.56 (*P* = 0.71)
Factors associated with depressive predominant polarity (versus hypomanic/manic predominant polarity)							
History of suicide attempts	10	1793	OR = 2.09 [1.49 to 2.93]	SMD = 0.41 [0.22 to 0.59]	0.001	45.2%	coeff.: −0.64 (*P* = 0.67)
Depressive polarity of first episode	9	1655	OR = 12.09 [6.38 to 22.90]	SMD = 1.37 [1.02 to 1.73]	0.001	78.3%	N/A
Number of mood episodes	9	1773	WMD = 0.99 episodes [0.28 to 1.70 episodes]	SMD = 0.12 [0.02 to 0.22]	0.006	0%	N/A
In a relationship	6	856	OR = 1.98 [1.22 to 3.22]	SMD = 0.38 [0.11 to 0.65]	0.006	28.9%	N/A

*k*, number of included studies; *N*, number of study participants; N/A, not applicable; OR, odds ratio; SMD, standardised mean difference; WMD, weighted mean difference.

**Table 4:** Factors associated with hypomanic/manic or depressive predominant polarity: quality of evidence

Variable	Consistency	Precision	Publication bias	Magnitude	Evidence
Factors associated with hypomanic/manic predominant polarity (versus depressive predominant polarity)					
Age (years)	↓	=	=	=	Moderate ⊕⊕⊕○
Male gender	=	=	=	=	High ⊕⊕⊕⊕
Age at onset (years)	=	=	=	=	High ⊕⊕⊕⊕
Manic first polarity	↓	=	↓	↑	Moderate ⊕⊕⊕○
Bipolar-I-disorder	↓	↓	=	↑	Moderate ⊕⊕⊕○
Psychotic features	↓	↓	=	=	Low ⊕⊕○○
Factors associated with depressive-predominant polarity (versus hypomanic/manic predominant polarity)					
History of suicide attempts	=	=	=	=	High ⊕⊕⊕⊕
Depressive first polarity	↓	=	↓	↑	Moderate ⊕⊕⊕○
Number of mood episodes	=	=	↓	=	Moderate ⊕⊕⊕○
In a relationship	=	↓	↓	=	Low ⊕⊕○○

↓, downgrade by one level; **=**, no upgrade/downgrade; ↑, upgrade by one level.

#### Age

A meta-analysis based on 18 studies including a total of 2827 participants showed that individuals with mPP were younger than those with dPP (WMD = −3.19 years, 95% CI: −5.30 to −1.08 years; *P* = 0.003; Supplementary Fig. 4). The quality of evidence was moderate, as although the meta-analysis was inconsistent (*I*^2^ = 74.3%), it produced a small but precise estimate (equivalent effect size: SMD = −0.26, 95% CI: −0.43 to −0.08) with a low risk of publication bias (Egger's coefficient: −0.99, *P* = 0.43).

#### Gender

Based on a meta-analysis of 19 studies with 3335 total participants, we found that people with mPP were more likely to be male (odds ratio = 1.39, 95% CI: 1.10 to 1.76; *P* = 0.005; Supplementary Fig. 5). The overall quality of evidence was high, considering the low between-study heterogeneity (*I*^2^ = 45.9%), the precision of findings (equivalent effect size: SMD = 0.18, 95% CI: 0.05 to 0.31) and the low risk of publication bias (Egger's coefficient: −0.28, *P* = 0.73).

#### Age at onset

Based on 13 studies including 2494 individuals with bipolar disorder, we estimated that people with mPP had earlier onset of bipolar disorder than people with dPP (WMD = −1.57 years, 95% CI: −2.88 to −0.26 years; *P* = 0.019; Supplementary Fig. 6). The overall quality of evidence was high, considering the low between-study heterogeneity (*I*^2^ = 42.3%), the precision of findings (equivalent effect size: SMD = −0.15, 95% CI: −0.27 to −0.02) and the low risk of publication bias (Egger's coefficient: 0.44, *P* = 0.69).

#### Manic polarity of first episode

We found that people with mPP were more likely to report a first mood episode characterised by manic polarity (*k* = 8, *N* = 1557; odds ratio = 13.54, 95% CI: 5.83 to 31.46; *P* < 0.001; Supplementary Fig. 7). The overall quality of evidence was moderate; despite the inconsistency of findings (*I*^2^ = 84.8%) and the unclear risk of publication bias, the magnitude of the effect and the lower and upper confidence limits (equivalent effect size: SMD = 1.44, 95% CI: 0.97 to 1.90) were large.

#### Bipolar-I disorder

The meta-analysis (*k* = 12; *N* = 1661) showed that people with mPP were more often affected by BD-I than individuals with dPP (odds ratio = 4.82, 95% CI: 2.27 to 10.24; *P* < 0.001; Supplementary Fig. 8), with a large effect (equivalent effect size: SMD = 0.87, 95% CI: 0.45 to 1.28). The evidence was of moderate quality, given the low precision of findings and the inconsistency (*I*^2^ = 81.8%), despite a low risk of publication bias (Egger's coefficient: 1.08, *P* = 0.54).

#### Psychotic features

Based on ten studies including 1970 participants with bipolar disorder, those with mPP were more likely to have psychotic features than those with dPP (odds ratio = 1.56, 95% CI: 1.01 to 2.41; *P* = 0.047; Supplementary Fig. 9). However, the overall evidence was of low quality, because of the moderate-to-high heterogeneity (*I*^2^ = 66.1%) and imprecision (equivalent effect size: SMD = 0.25, 95% CI: 0.01 to 0.49), regardless of the low risk of publication bias (Egger's coefficient: −0.56, *P* = 0.71).

### Variables associated with depressive predominant polarity

A summary of findings is provided in Table 3, and related details on the quality of evidence are given in Table 4.

#### History of suicide attempts

Meta-analysis based on ten studies and 1793 subjects showed that people with dPP were more likely to have attempted suicide (odds ratio = 2.09, 95% CI: 1.49 to 2.93; *P* < 0.001; Supplementary Fig. 10). The quality of evidence was high in terms of consistency (*I*^2^ = 45.2%), precision (equivalent effect size: SMD = 0.41, 95% CI: 0.22 to 0.59) and risk of publication bias (Egger's coefficient: −0.64, *P* = 0.67).

#### Depressive polarity of first episode

We found that a depressive polarity of the first mood episode was associated with dPP (*k* = 9, *N* = 1655; odds ratio = 12.09, 95% CI: 6.38 to 22.90; *P* < 0.001; Supplementary Fig. 11). The evidence was of moderate quality, considering the poor consistency across studies (*I*^2^ = 78.3%) and the unclear risk of publication bias, despite the large effect (equivalent effect size: SMD = 1.37, 95% CI: 1.02 to 1.73).

#### Number of mood episodes

Individuals with dPP had more mood episodes than people with mPP (k = 9; *N* = 1773; WMD = 0.99 mood episodes; 95% CI: 0.28 to 1.70 mood episodes; *P* = 0.006; Supplementary Fig. 12). Despite the consistency across studies (*I*^2^ = 0%) and the precision of findings (equivalent effect size: SMD = 0.12, 95% CI: 0.02 to 0.22), the evidence was of moderate quality, being downgraded by one level because of uncertainty about publication bias.

#### Being in a relationship

Meta-analysis based on six studies and 856 participants showed that people with dPP were more often married or in a relationship than those with mPP (odds ratio = 1.98, 95% CI: 1.22 to 3.22; *P* = 0.006; Supplementary Fig. 13). However, the quality of evidence was low, owing to the poor precision of findings (equivalent effect size: SMD = 0.38, 95% CI: 0.11 to 0.65) and the unclear risk of publication bias, despite the low between-study heterogeneity (*I*^2^ = 28.9%).

### Variables not associated with any predominant polarity

We found no differences between mPP and dPP as regards other variables, including years of education, unemployment, duration of illness, mixed polarity of first episode, rapid cycling course, number of hospital admissions, number of suicide attempts, comorbid alcohol and substance use disorders, and family history of bipolar disorder, any affective disorders or suicide. A summary of these findings is provided in Supplementary Table 1, and relevant forest plots are shown in Supplementary Figs. 14–25.

## Discussion

### Summary and interpretation of findings

To our knowledge, this is the first systematic review and meta-analysis investigating prevalence rates and possible correlates of mPP and dPP in bipolar disorder. Based on 28 studies, conducted in 12 countries across Europe, Asia and South America, our work provides several important findings.

First, we found no differences in prevalence rates between predominant polarities, as around a third of subjects with bipolar disorder had mPP, and a similar proportion of individuals had dPP. Thus, a predominant polarity, as defined by Colom's criteria,^11^ seems to affect approximately two-thirds of patients with bipolar disorder. Overall rates of both mPP and dPP as estimated in our work appeared higher than those reported in a previous review on this topic, which found that around half of people with bipolar disorder might have a well-defined predominant polarity.^8^

Second, rates of mPP and dPP might vary according to different geographical areas. Whereas dPP was more frequent than mPP in European countries, opposite estimates were found in studies conducted in Asia, in which prevalence rates of mPP were almost double those of dPP. This was not surprising, as the clinical trajectory of bipolar disorder might be influenced by genetic, cultural and environmental factors that are likely to vary by country,^53^ shaping the expression of manic and depressive symptoms. Moreover, differences in predominant polarity might be explained also by cross-national variations in prevalence rates of BD-I and BD-II,^54^ which are associated with mPP and dPP, respectively. Finally, it should be considered that even the heterogeneity of mental healthcare delivery systems worldwide may influence the probability of access to care for patients with bipolar disorder, especially during depressive episodes.^55,56^ Indeed, fewer than half of people with bipolar disorder receive mental health treatment, particularly in low-income countries, and only a quarter report contacts with the mental health system.^57^ However, those with manic episodes and related behavioural abnormalities may be less likely to avoid detection and treatment.

Third, we uncovered several clinically meaningful correlates associated with a distinct predominant polarity. Individuals with mPP were younger, more often male and more likely to be affected by BD-I, as well as being more likely to show psychotic features and have an earlier onset characterised by manic symptoms. These findings were consistent with evidence from a recently published meta-analysis involving participants whose manic predominance was set by definition, i.e. those with unipolar mania, who were again more often males, with younger age at onset and higher rates of psychotic features.^28^ In addition, our findings, showing an association between mPP and BD-I, seem consistent with the natural history of BD-I,^58^ which is typically characterised by more psychotic features and an earlier onset than BD-II. On the other hand, people with dPP were more likely to have a depressive polarity at onset, a higher number of mood episodes and a history of suicide attempts, and were more often in a relationship than people with mPP. Our findings seem to be supported by a relatively recent body of evidence in the field.^1,59^

A balanced interpretation of our findings needs to consider that for some explored variables, the quality of evidence was low (i.e. psychotic features and being in a relationship) or moderate (i.e. age, polarity of first episode, BD-I and number of mood episodes). This was influenced by several issues, including the imprecision and inconsistency of overall estimates, as well as the uncertain probability of publication bias. Additional research is needed to substantiate our meta-analytic evidence for these variables. Nonetheless, our findings reasonably support the hypothesis that predominant polarity might identify specific subgroups of people with bipolar disorder; in particular, the associations with male gender and earlier bipolar disorder onset in mPP and with higher rates of suicide attempts in dPP were based on evidence of high quality.

### Clinical implications

The assessment and definition of predominant polarity, because of its potential as a long-term specifier, may represent a valuable tool in clinical practice to help choose appropriate treatments and predict probable outcomes for individuals with bipolar disorder. In particular, the predominant polarity conceptualisation might represent a useful alternative to the traditional and yet complex^60,61^ distinction between BD-I and BD-II.^9^ In view of the existing scientific evidence in this field, the inclusion of predominant polarity in the treatment decision-making process for bipolar disorder has been already hypothesised, with the concept of the polarity index.^9,13^ This defines the ratio between antimanic and antidepressant properties of single pharmacological and non-pharmacological approaches used for maintenance treatment of bipolar disorder.^62,63^ More generally, establishing clinical features associated with mPP or dPP may guide clinicians in the early identification of possible trajectories of bipolar disorder and in the selection of the most appropriate interventions for the prevention of mood relapses. In addition, the conceptualisation of predominant polarity, involving a fine-grained assessment of the behaviour, cognition, emotions and social interactions of individuals with bipolar disorder, might clash with emerging perspectives towards an unified and transdiagnostic view of affective disorders.^64^

Another important clinical implication of this study is related to the strong concordance between the long-term predominant polarity and the polarity of the first mood episode: our meta-analyses showed large effects for the relationships between a manic onset and mPP, as well as between a depressive onset and dPP, ruling out any association between mixed symptoms at onset and subsequent predominant polarity. While stressing the clinical heterogeneity of mixed features possibly occurring in both manic and depressive episodes,^65^ our findings make clearer the predictive role of the first mood episode on predominant polarity. This has also been suggested by some pioneering studies in the field,^66,67^ which showed that affective polarity at onset was associated with the polarity of the following episodes. As the main critical element of the current conceptualisation of predominant polarity involves the large amount of time that elapses between onset of bipolar disorder and the subsequent assessment of mPP, dPP or undefined polarity, across at least three mood episodes,^8^ the first manic or depressive episode might be considered to be a useful clinical proxy for the polarity of subsequent recurrences.

Finally, from a completely different perspective, the definition of potential neurobiological correlates of mPP and dPP might represent an important area for future research. Recent studies have reported, for example, that individuals with predominant polarity – especially those with mPP – may show differences in cortical thickness in several areas of the central nervous system.^31,35^ Further evidence highlighted that biomarkers of bipolar disorder may be more influenced by its specific clinical features, e.g. depression and mania, than by the disease itself.^68–70^ Potential neurobiological underpinnings of predominant polarity in bipolar disorder are also a matter for future research.

### Limitations

The findings of the current systematic review and meta-analysis should be interpreted with caution, considering some limitations. First, as our work investigated cross-sectional differences between mPP and dPP, we cannot draw any conclusions about causal inference. Second, we need to consider some methodological variability across studies in terms of objectives, sample size, inclusion criteria and methods used to assess clinical variables. For instance, the retrospective nature of data should be considered as a possible source of misclassification of predominant polarity. Third, owing to the observational nature of the included studies, without any predefined protocol, we should consider that the possible effects of unpublished data and the likelihood of selective reporting bias may have at least partially influenced our meta-analytic estimates. Moreover, considering the lack of valid and recommended tools for meta-analyses based on non-intervention and non-randomised studies,^71^ we did not formally assess the risk of bias of included studies. Although we included only studies with consistent definitions of predominant polarity, the poor representativeness of some studies (e.g. those including only in-patients) and some risk of recall bias (due to the nature of the tested variables) may have affected the results of our meta-analysis. In addition, the clinical course of bipolar disorder may be influenced by the different effectiveness of treatments for preventing relapses; however, insufficient data were available from the included studies for us to explore the role of this confounder on polarity predominance. For example, lithium, the gold-standard treatment for bipolar disorder,^72^ has shown to be more effective in preventing mania than depression,^73^ and, in general, there are fewer evidence-based treatments for bipolar depression than for mania.^74^ Similarly, data on other important correlates, such as seasonality,^75^ affective temperaments^32^ and comorbid anxiety disorders,^17^ were available only from a limited number of studies, suggesting a need for additional research.

### Future perspectives

The findings of this systematic review and meta-analysis support the hypothesis that predominant polarity might be a useful specifier of bipolar disorder, identifying subgroups of individuals with different clinical characteristics. High quality of evidence shows an association of mPP with male gender and an earlier onset of bipolar disorder, whereas dPP is more often associated with a history of suicide attempts. Different predominant polarities in bipolar disorder may represent particular targets for more appropriate care programmes and effective approaches to personalised treatment.

## Supporting information

Bartoli et al. supplementary material 1 Bartoli et al. supplementary material

Bartoli et al. supplementary material 2 Bartoli et al. supplementary material

## Data Availability

The data that support the findings of this study are available from the corresponding author (F.B.) upon reasonable request.
