# Supplementary material for: Prevalence and correlates of manic/hypomanic and depressive predominant polarity in bipolar disorder: systematic review and meta-analysis
Source: BJPsych Open. 2024 May 6;10(3):e100. doi: 10.1192/bjo.2024.51 (PMC11094450; doi:10.1192/bjo.2024.51)

**Supplementary Figures**

[Prevalence of hypomanic/manic and depressive predominant polarity in bipolar disorder 1](#_Toc152149082)

[Supplementary Figure 1. Weighted prevalence of hypomanic/manic predominant polarity in bipolar disorder. 1](#_Toc152149083)

[Supplementary Figure 2. Weighted prevalence of depressive predominant polarity in bipolar disorder. 2](#_Toc152149084)

[Supplementary Figure 3. Weighted prevalence difference of hypomanic/manic vs. depressive predominant polarity in bipolar disorder. 3](#_Toc152149085)

[Variables associated with hypomanic/manic predominant polarity 4](#_Toc152149086)

[Supplementary Figure 4. Mean difference in age (years) between participants with a hypomanic/manic predominant polarity and those with a depressive predominant polarity. 4](#_Toc152149087)

[Supplementary Figure 5. Male gender in participants with a hypomanic/manic predominant polarity vs. those with a depressive predominant polarity. 5](#_Toc152149088)

[Supplementary Figure 6. Mean difference in age at onset (years) between participants with a hypomanic/manic predominant polarity and those with a depressive predominant polarity. 6](#_Toc152149089)

[Supplementary Figure 7. Manic polarity of first episode in participants with a hypomanic/manic predominant polarity vs. those with a depressive predominant polarity. 7](#_Toc152149090)

[Supplementary Figure 8. Diagnosis of bipolar-I disorder in participants with a hypomanic/manic predominant polarity vs. those with a depressive predominant polarity. 8](#_Toc152149091)

[Supplementary Figure 9. Psychotic features in participants with a hypomanic/manic predominant polarity vs. those with a depressive predominant polarity. 9](#_Toc152149092)

[Variables associated with depressive predominant polarity 10](#_Toc152149093)

[Supplementary Figure 10. History of suicide attempts in participants with a depressive predominant polarity vs. those with a hypomanic/manic predominant polarity. 10](#_Toc152149094)

[Supplementary Figure 11. Depressive polarity of first episode in participants with a depressive predominant polarity vs. those with a hypomanic/manic predominant polarity. 11](#_Toc152149095)

[Supplementary Figure 12. Mean difference in number of mood episodes between participants with a depressive predominant polarity and those with a hypomanic/manic predominant polarity. 12](#_Toc152149096)

[Supplementary Figure 13. Being in a relationship in participants with a depressive predominant polarity vs. those with a hypomanic/manic predominant polarity. 13](#_Toc152149097)

[Variables not associated with any predominant polarity 14](#_Toc152149098)

[Supplementary Figure 14. Mean difference in years of education between participants with a hypomanic/manic predominant polarity and those with a depressive predominant polarity. 14](#_Toc152149099)

[Supplementary Figure 15. Unemployment in participants with a hypomanic/manic predominant polarity vs. those with a depressive predominant polarity. 15](#_Toc152149100)

[Supplementary Figure 16. Mean difference in duration of illness (years) between participants with a hypomanic/manic predominant polarity and those with a depressive predominant polarity. 16](#_Toc152149101)

[Supplementary Figure 17. Mixed polarity of episode in participants with a hypomanic/manic predominant polarity vs. those with a depressive predominant polarity. 17](#_Toc152149102)

[Supplementary Figure 18. Rapid cycling course in participants with a hypomanic/manic predominant polarity vs. those with a depressive predominant polarity. 18](#_Toc152149103)

[Supplementary Figure 19. Mean difference in number of hospital admission between participants with a hypomanic/manic predominant polarity and those with a depressive predominant polarity. 19](#_Toc152149104)

[Supplementary Figure 20. Mean difference in number of suicide attempts between participants with a hypomanic/manic predominant polarity and those with a depressive predominant polarity. 20](#_Toc152149105)

[Supplementary Figure 21. Alcohol use disorder in participants with a hypomanic/manic predominant polarity vs. those with a depressive predominant polarity. 21](#_Toc152149106)

[Supplementary Figure 22. Substance use disorder in participants with a hypomanic/manic predominant polarity vs. those with a depressive predominant polarity. 22](#_Toc152149107)

[Supplementary Figure 23. Family history of bipolar disorder in participants with a hypomanic/manic predominant polarity vs. those with a depressive predominant polarity. 23](#_Toc152149108)

[Supplementary Figure 24. Family history of affective disorders in participants with a hypomanic/manic predominant polarity vs. those with a depressive predominant polarity. 24](#_Toc152149109)

[Supplementary Figure 25. Family history of suicide in participants with a hypomanic/manic predominant polarity vs. those with a depressive predominant polarity. 25](#_Toc152149110)

#

# Prevalence of hypomanic/manic and depressive predominant polarity in bipolar disorder

## **Supplementary Figure 1.** Weighted prevalence of hypomanic/manic predominant polarity in bipolar disorder.


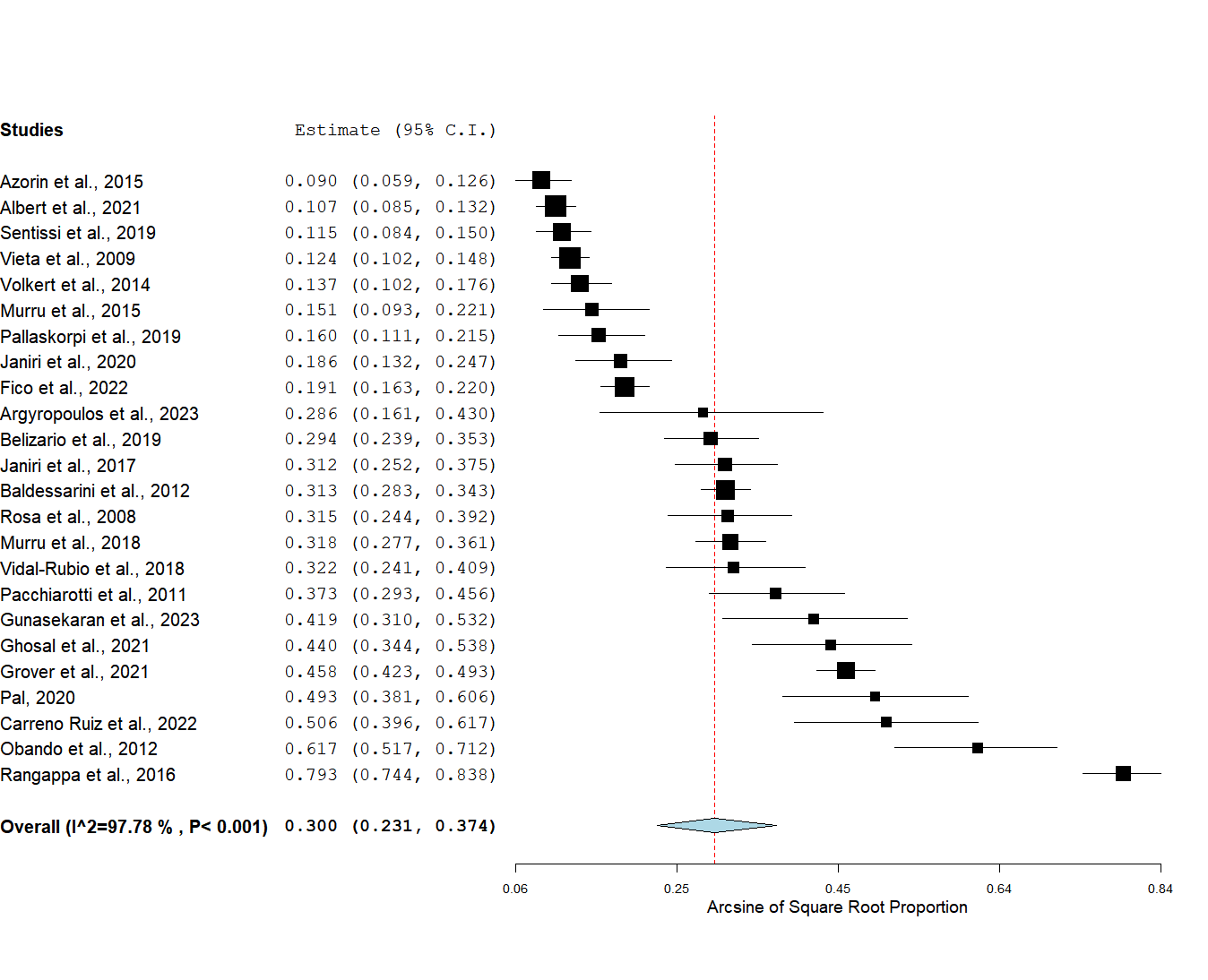


## **Supplementary Figure 2.** Weighted prevalence of depressive predominant polarity in bipolar disorder.


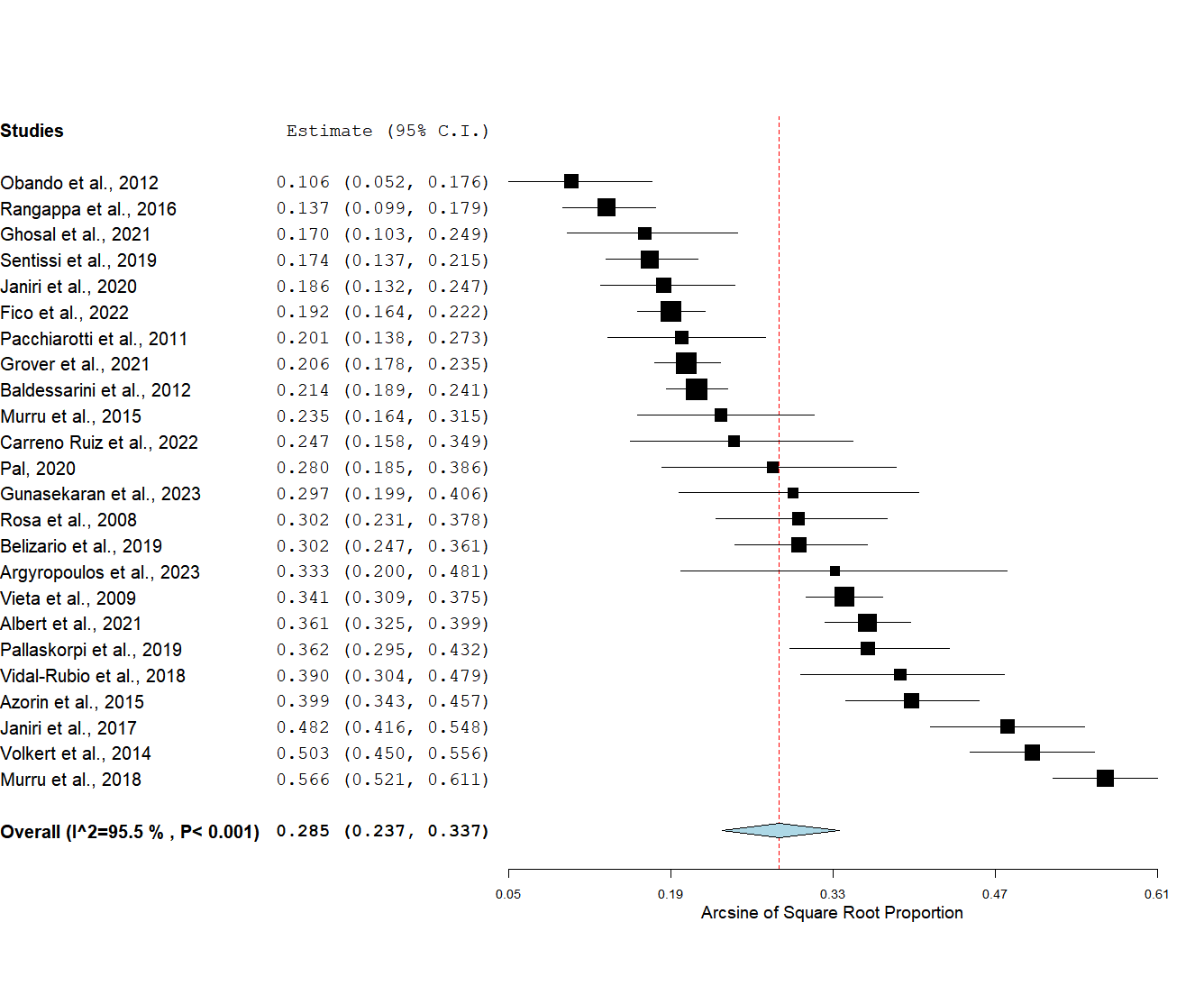


## **Supplementary Figure 3.** Weighted prevalence difference of hypomanic/manic vs. depressive predominant polarity in bipolar disorder.


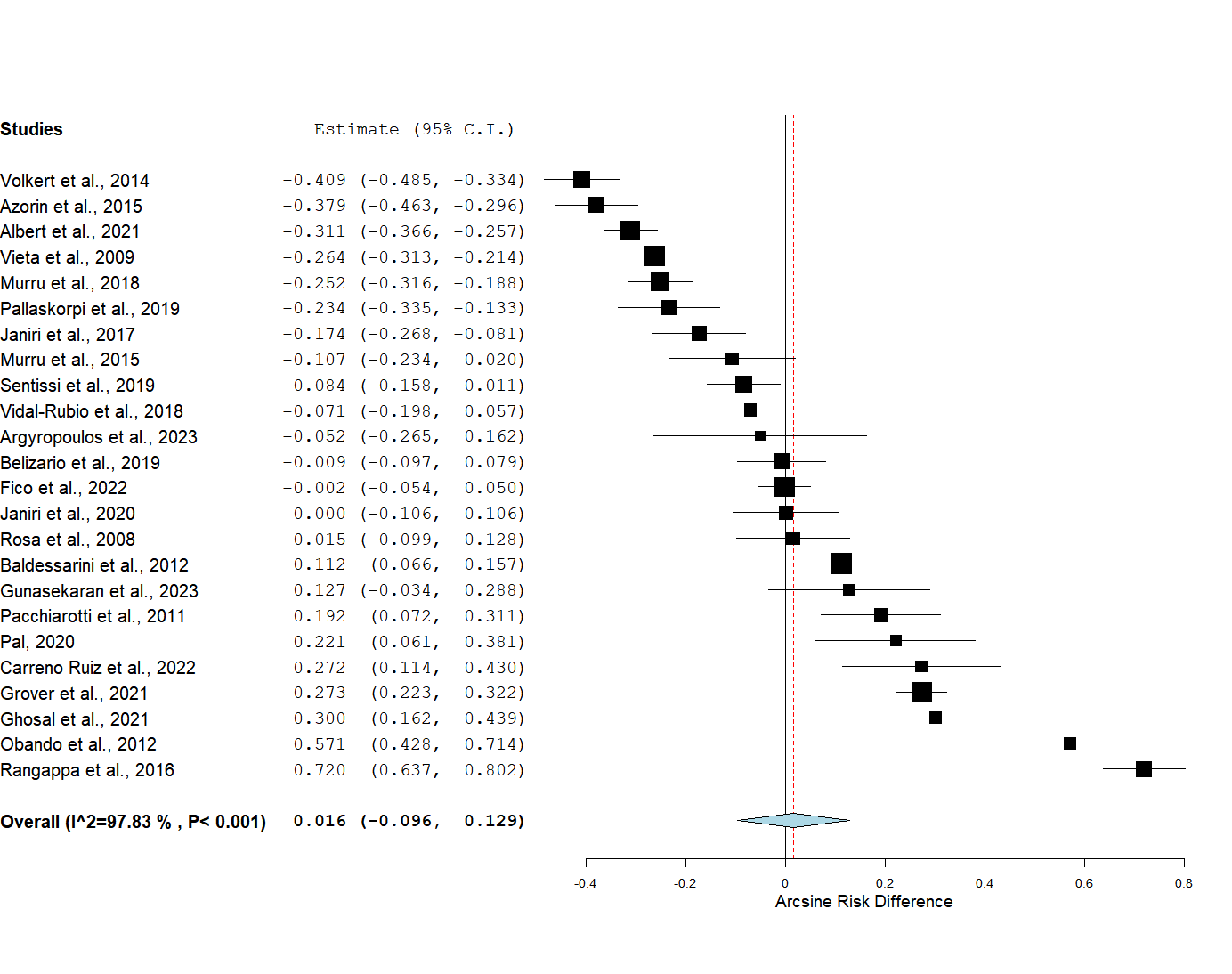


# Variables associated with hypomanic/manic predominant polarity

## **Supplementary Figure 4.** Mean difference in age (years) between participants with a hypomanic/manic predominant polarity and those with a depressive predominant polarity.


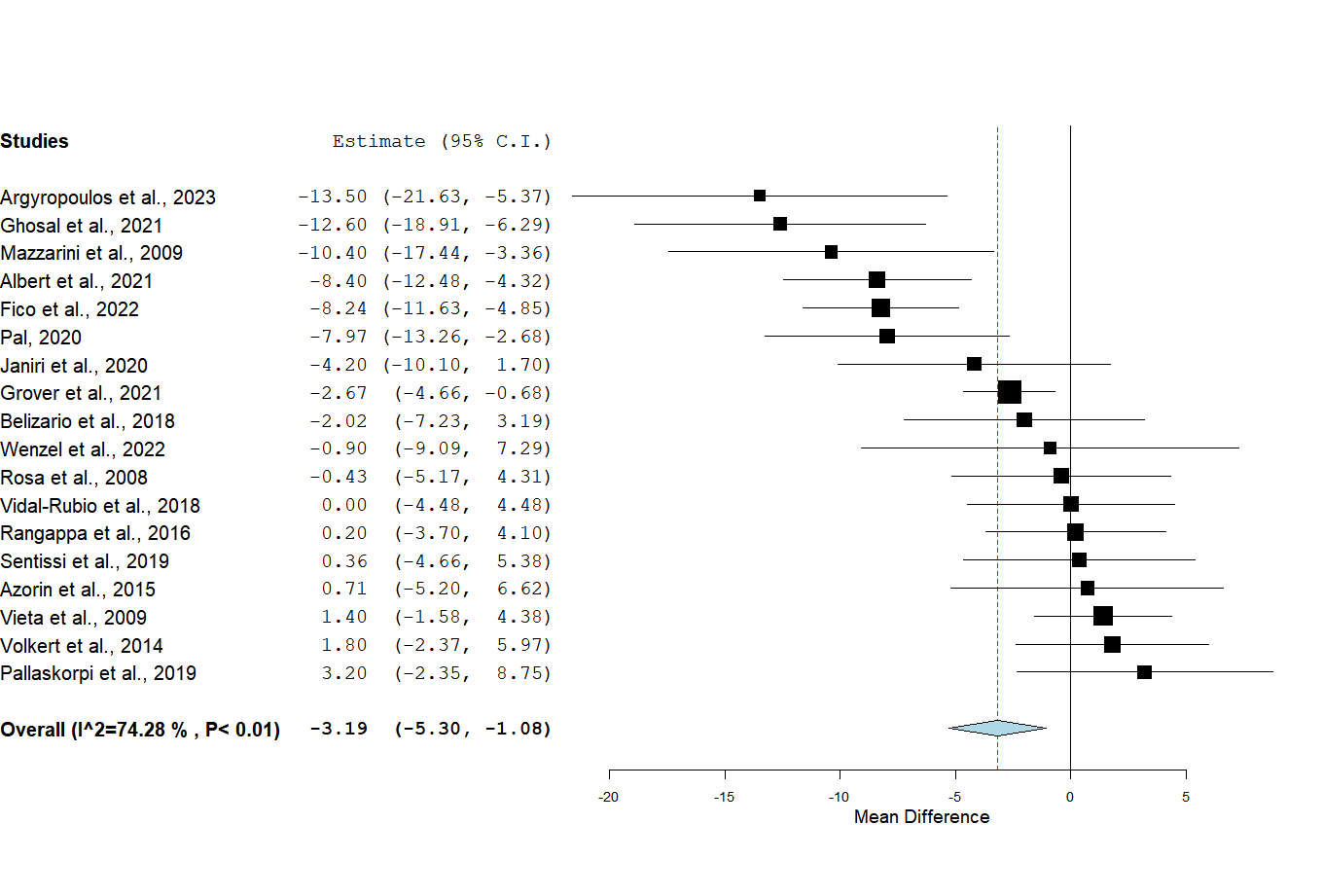


## **Supplementary Figure 5.** Male gender in participants with a hypomanic/manic predominant polarity vs. those with a depressive predominant polarity.


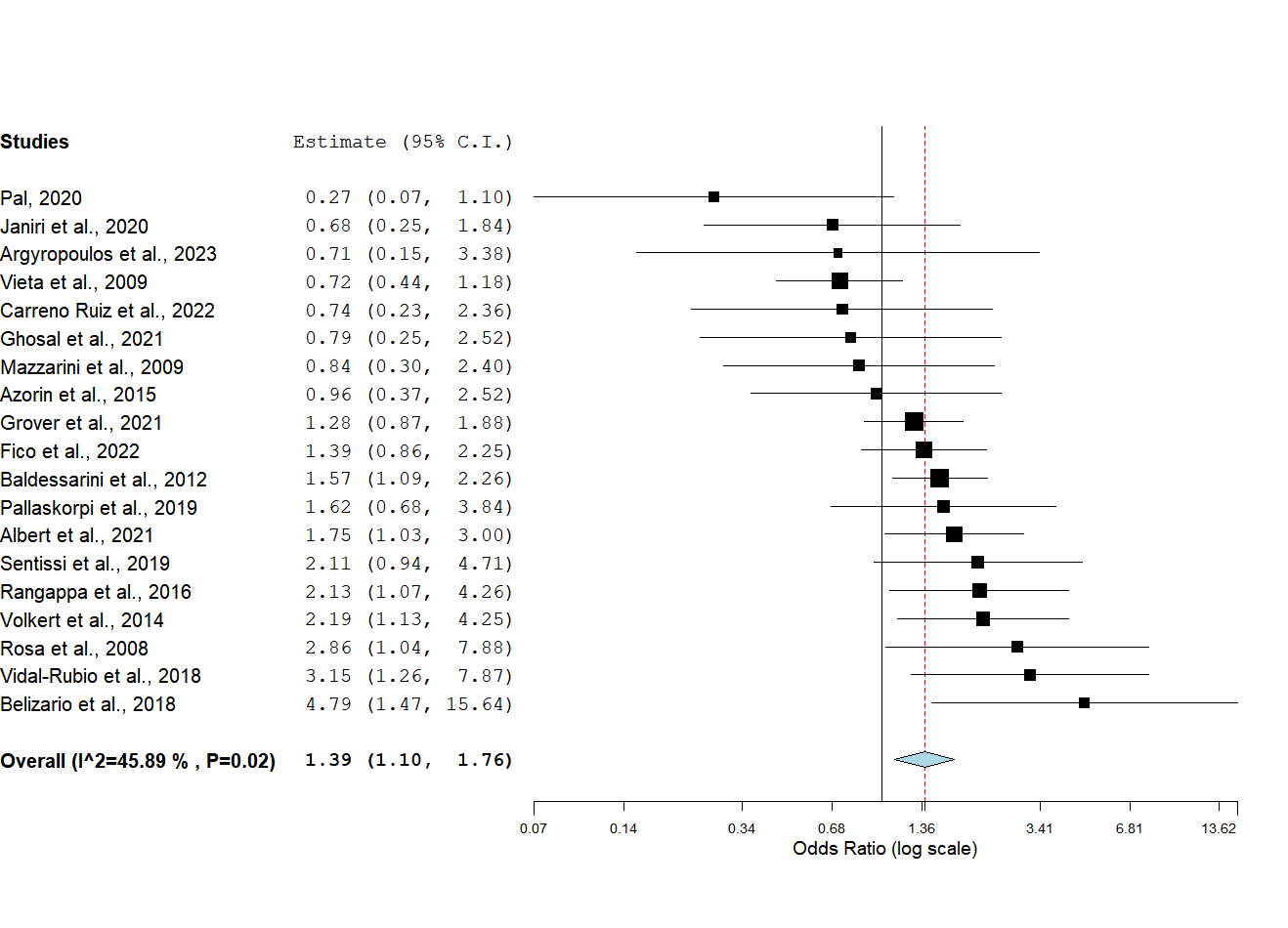


## **Supplementary Figure 6.** Mean difference in age at onset (years) between participants with a hypomanic/manic predominant polarity and those with a depressive predominant polarity.


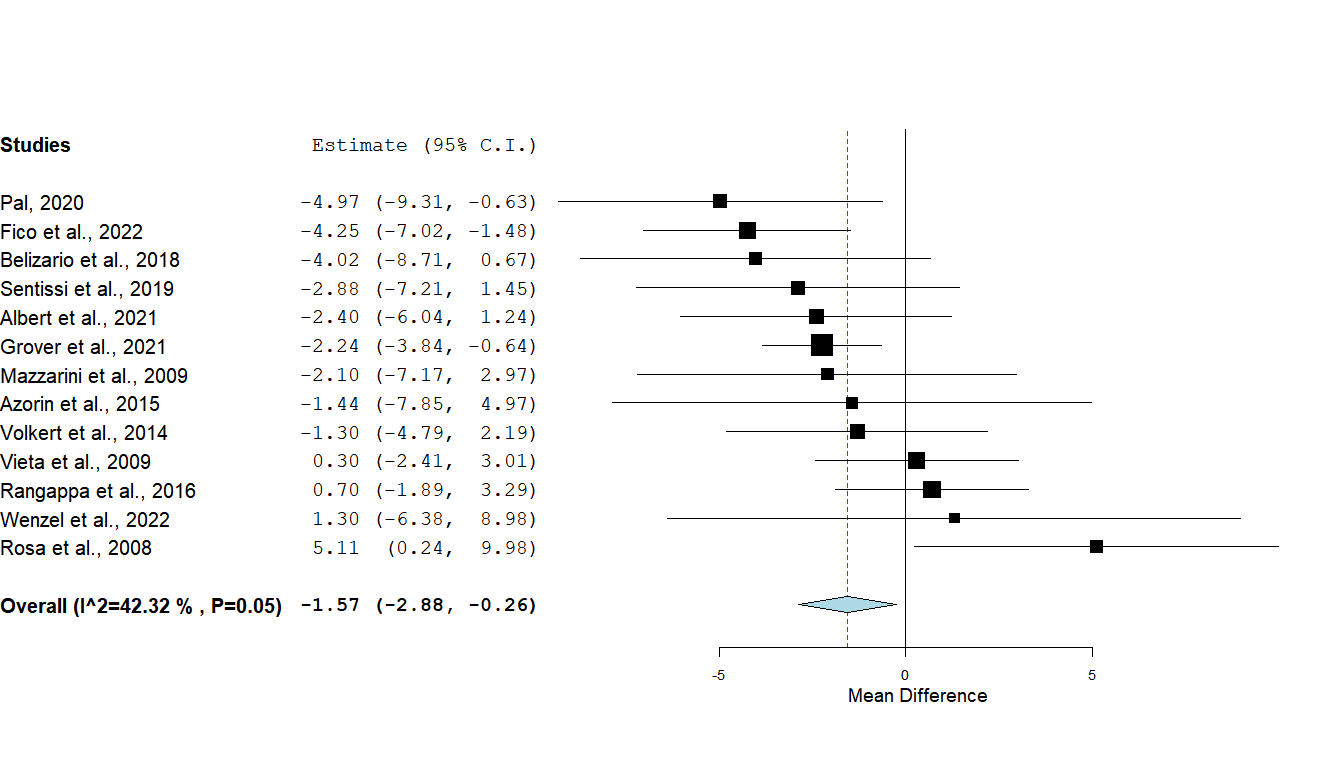


## **Supplementary Figure 7.** Manic polarity of first episode in participants with a hypomanic/manic predominant polarity vs. those with a depressive predominant polarity.


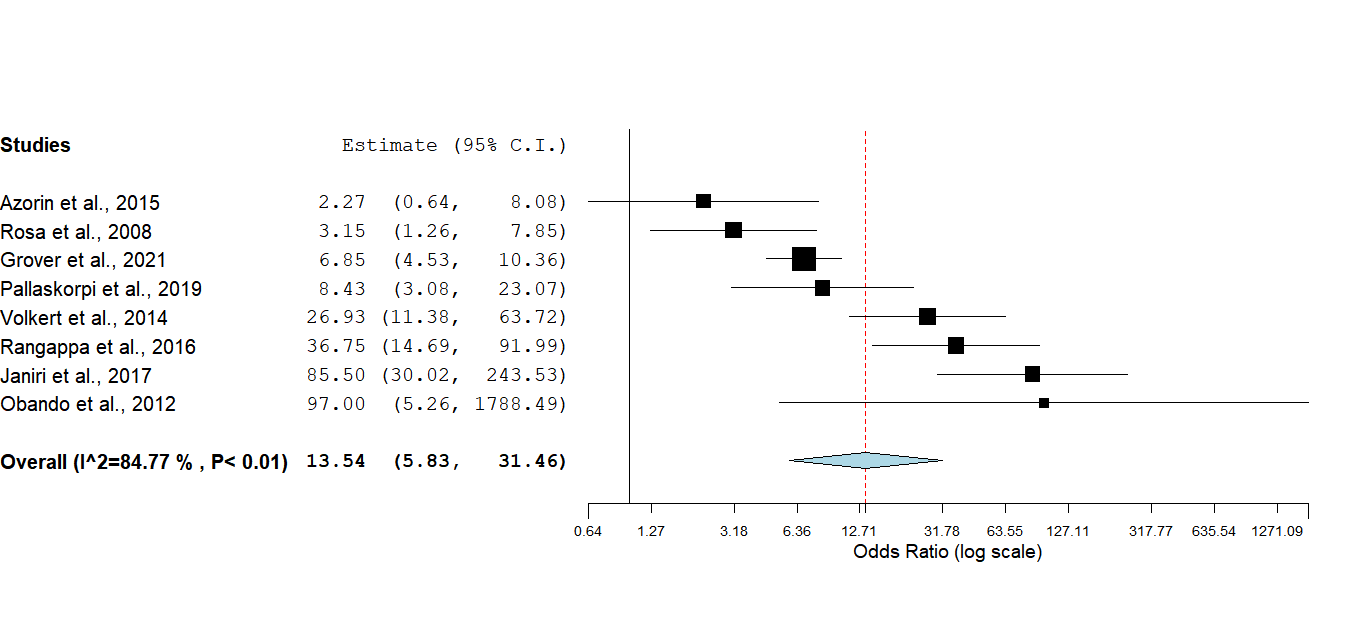


## **Supplementary Figure 8.** Diagnosis of bipolar-I disorder in participants with a hypomanic/manic predominant polarity vs. those with a depressive predominant polarity.


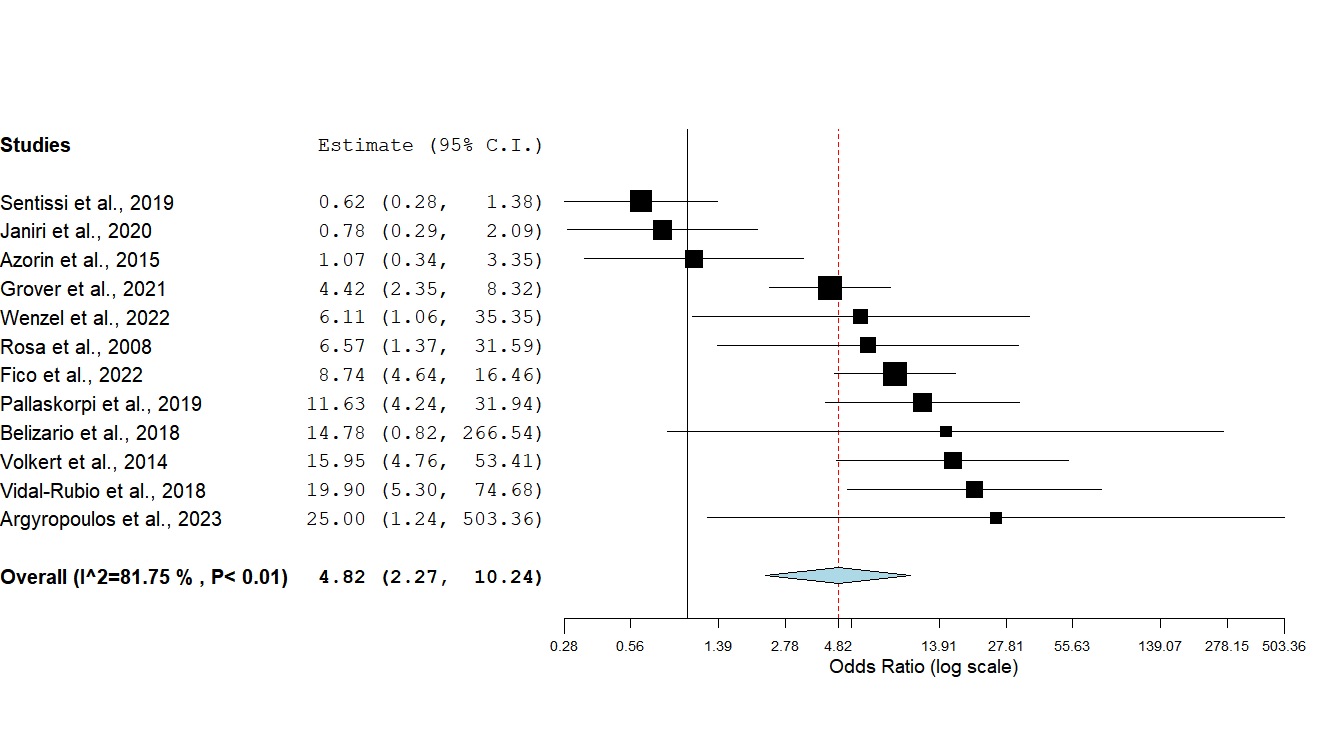


## **Supplementary Figure 9.** Psychotic features in participants with a hypomanic/manic predominant polarity vs. those with a depressive predominant polarity.


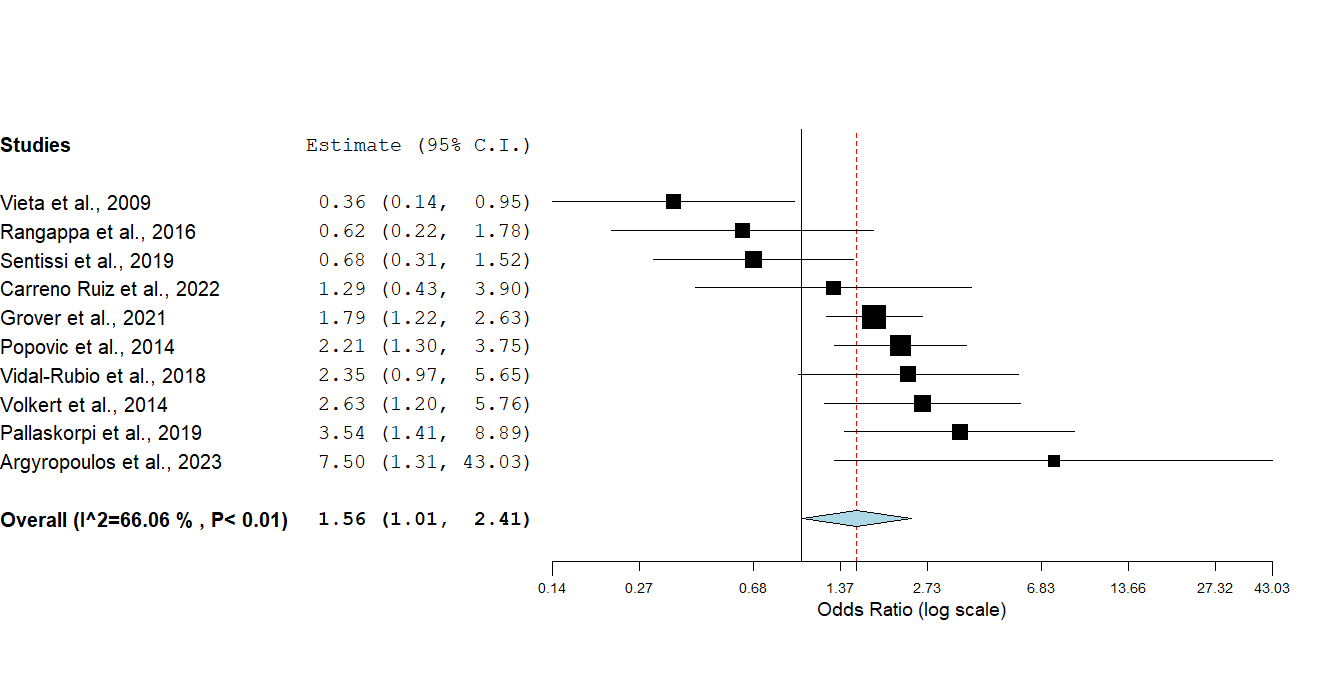


# Variables associated with depressive predominant polarity

## **Supplementary Figure 10.** History of suicide attempts in participants with a depressive predominant polarity vs. those with a hypomanic/manic predominant polarity.


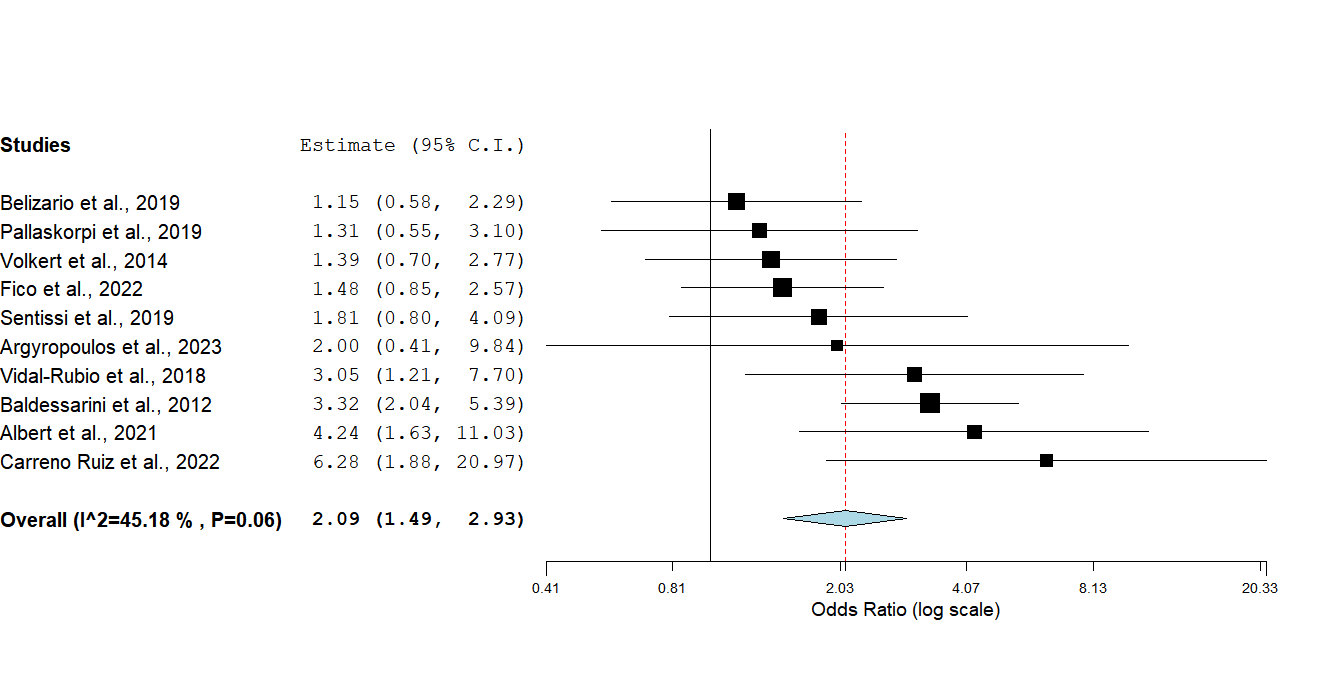


## **Supplementary Figure 11.** Depressive polarity of first episode in participants with a depressive predominant polarity vs. those with a hypomanic/manic predominant polarity.


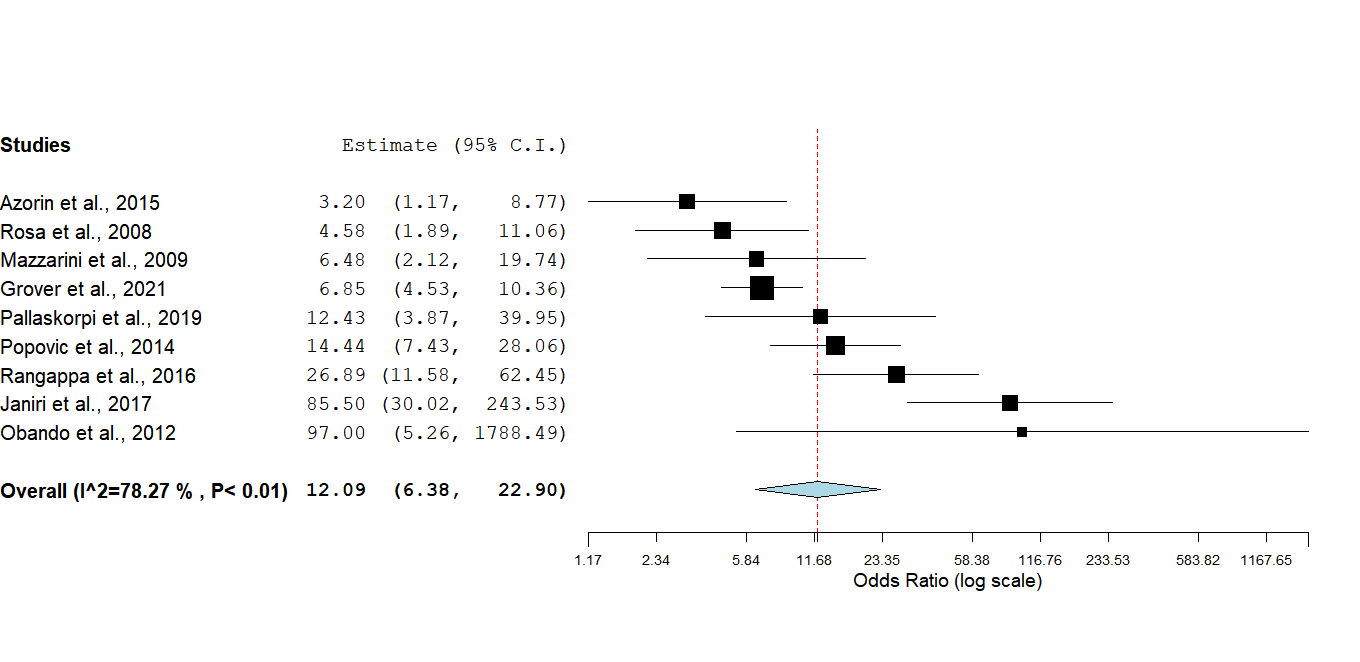


## **Supplementary Figure 12.** Mean difference in number of mood episodes between participants with a depressive predominant polarity and those with a hypomanic/manic predominant polarity.


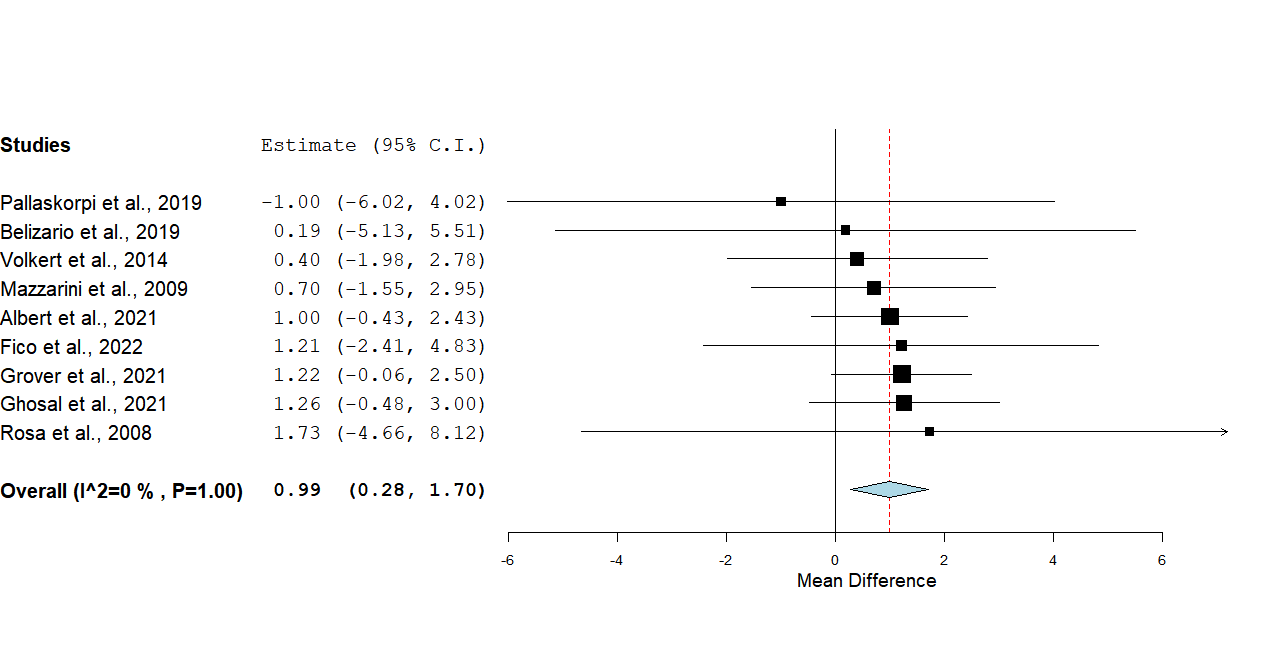


## **Supplementary Figure 13.** Being in a relationship in participants with a depressive predominant polarity vs. those with a hypomanic/manic predominant polarity.


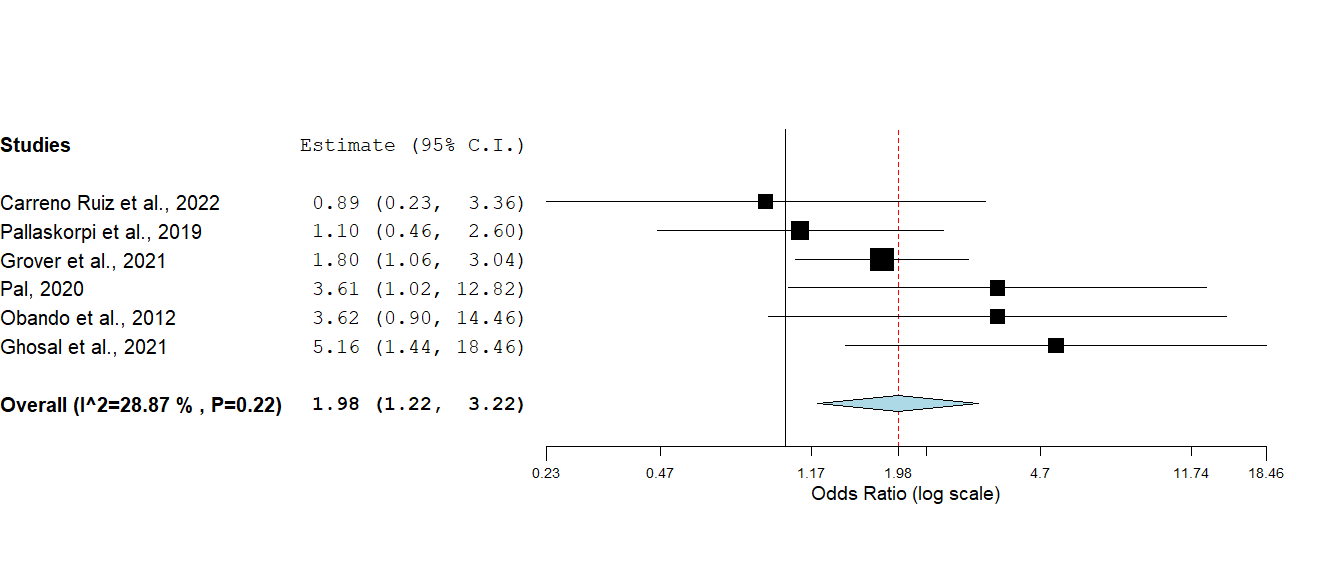


# Variables not associated with any predominant polarity

## **Supplementary Figure 14.** Mean difference in years of education between participants with a hypomanic/manic predominant polarity and those with a depressive predominant polarity.


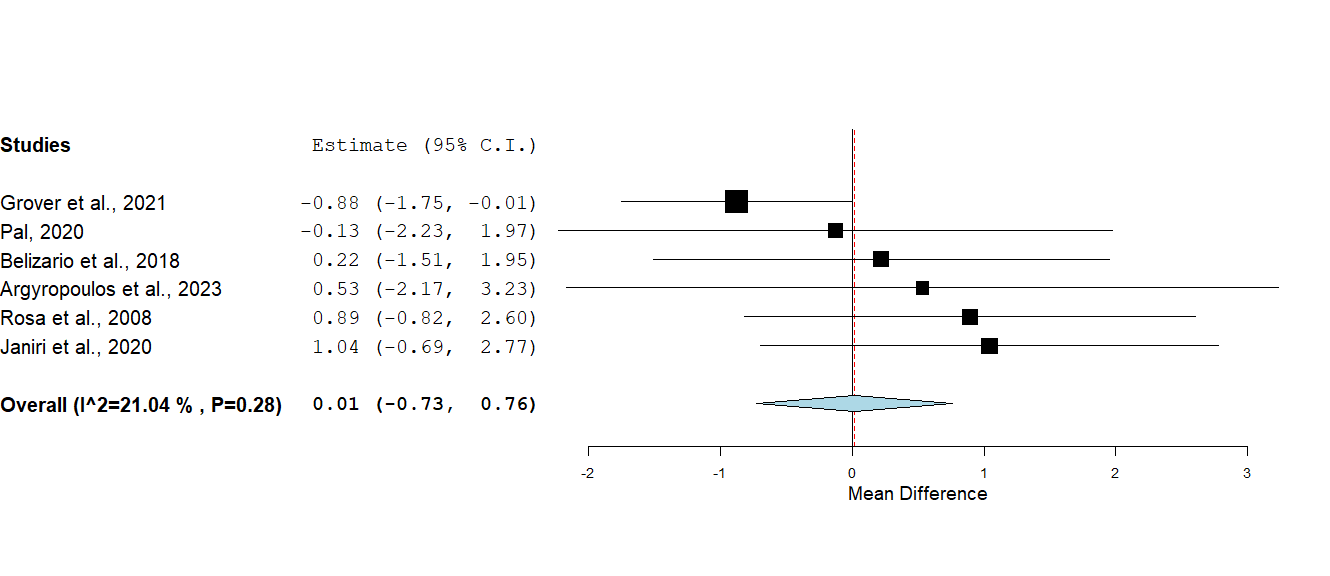


## **Supplementary Figure 15.** Unemployment in participants with a hypomanic/manic predominant polarity vs. those with a depressive predominant polarity.


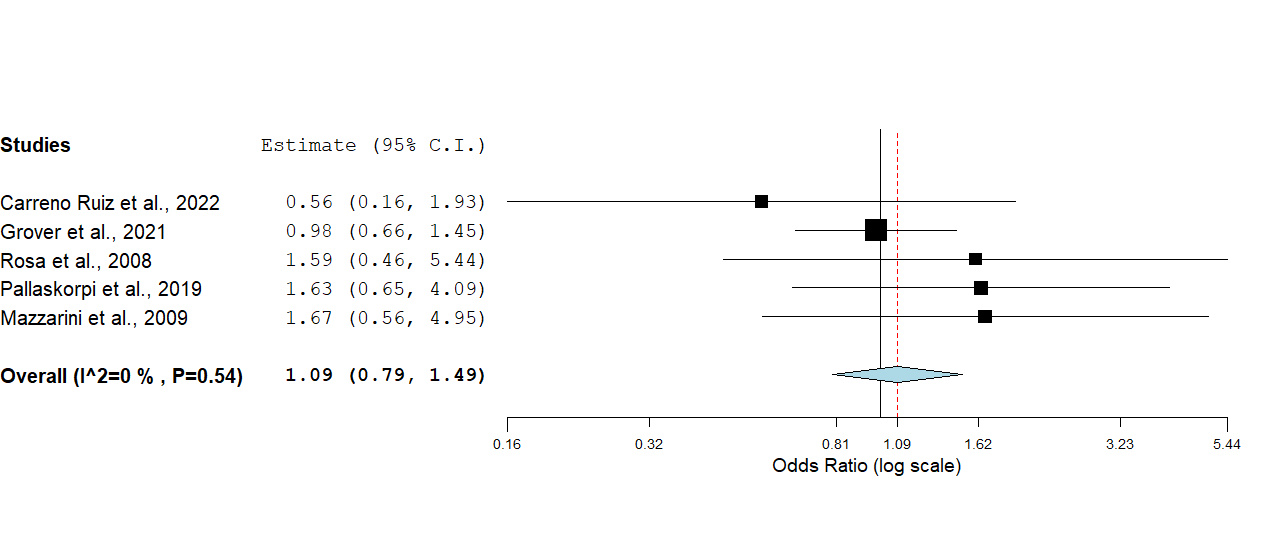


## **Supplementary Figure 16.** Mean difference in duration of illness (years) between participants with a hypomanic/manic predominant polarity and those with a depressive predominant polarity.


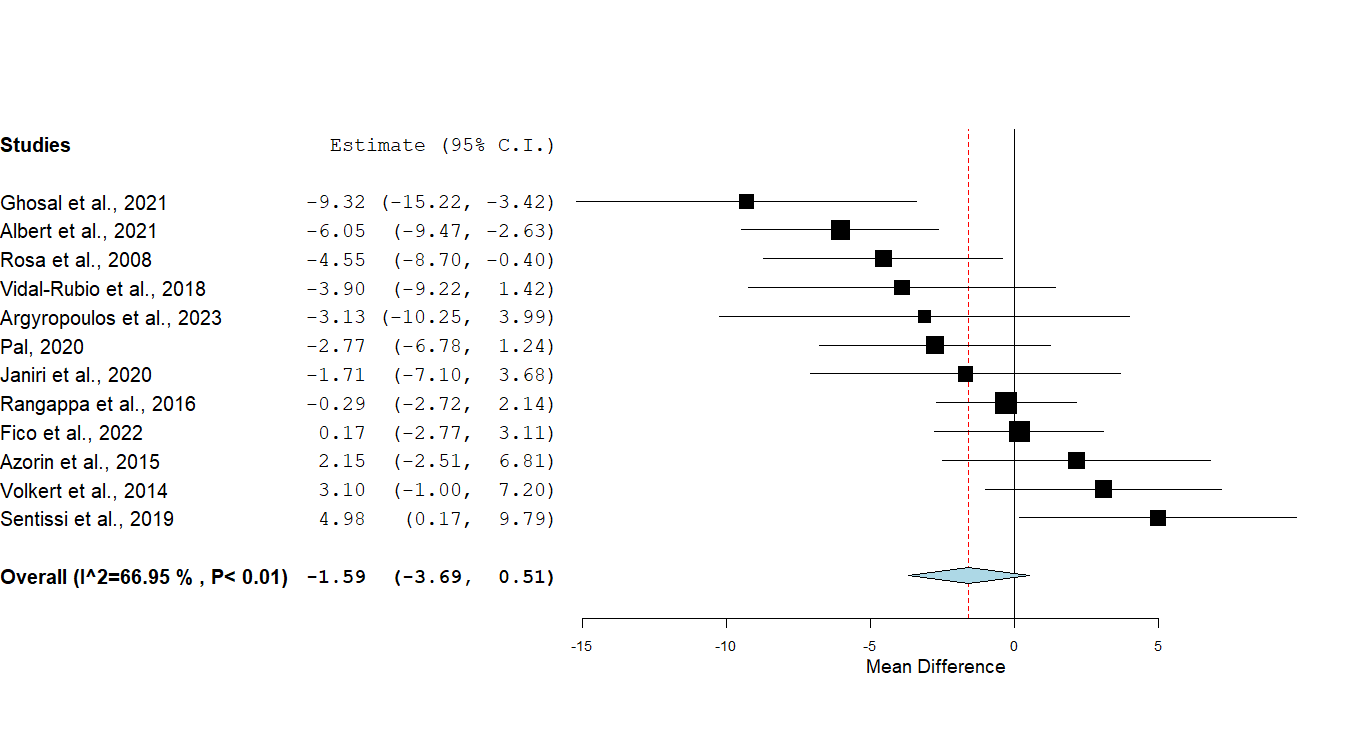


## **Supplementary Figure 17.** Mixed polarity of episode in participants with a hypomanic/manic predominant polarity vs. those with a depressive predominant polarity.


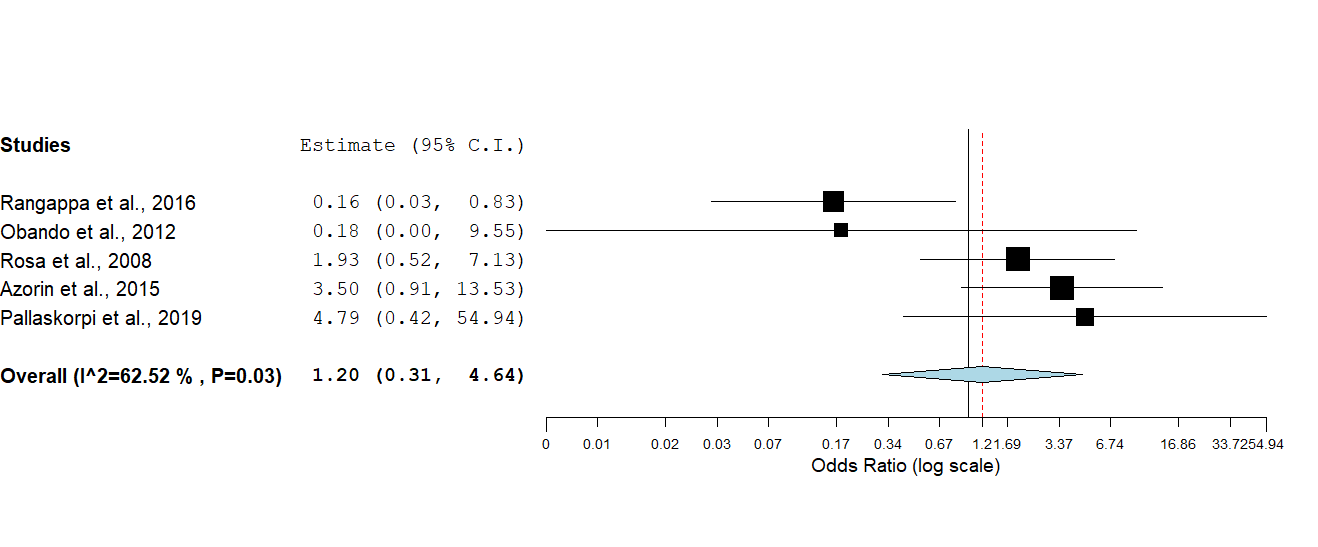


## **Supplementary Figure 18.** Rapid cycling course in participants with a hypomanic/manic predominant polarity vs. those with a depressive predominant polarity.


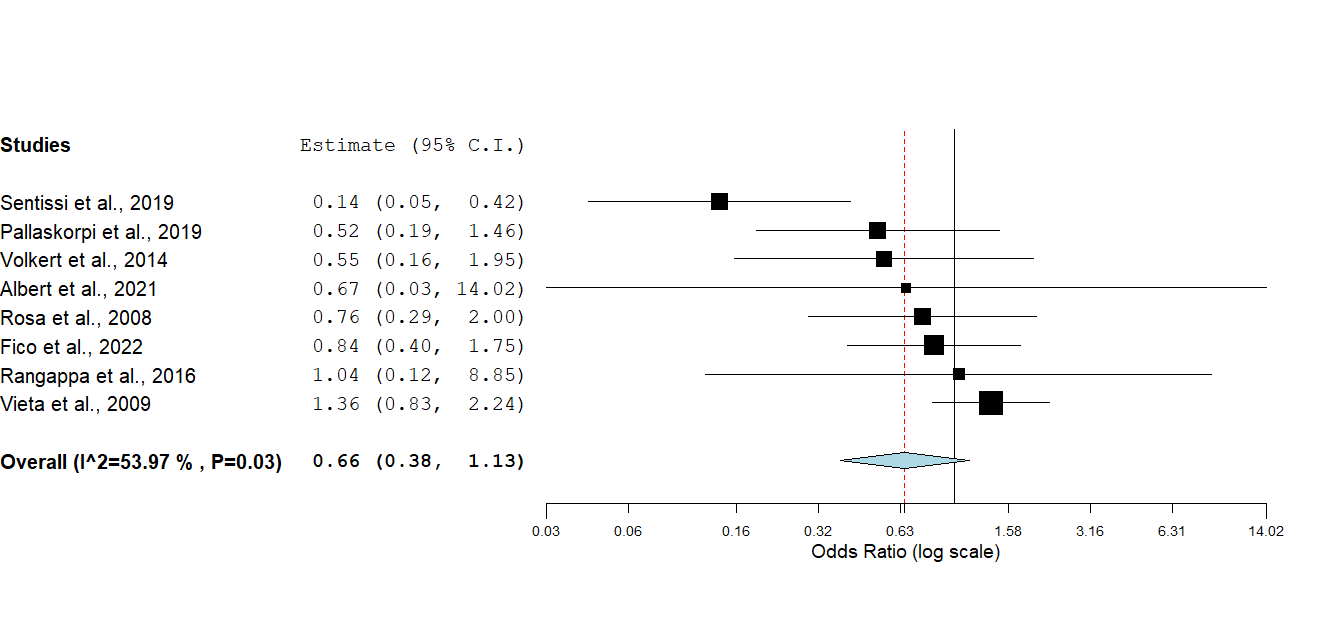


## **Supplementary Figure 19.** Mean difference in number of hospital admission between participants with a hypomanic/manic predominant polarity and those with a depressive predominant polarity.


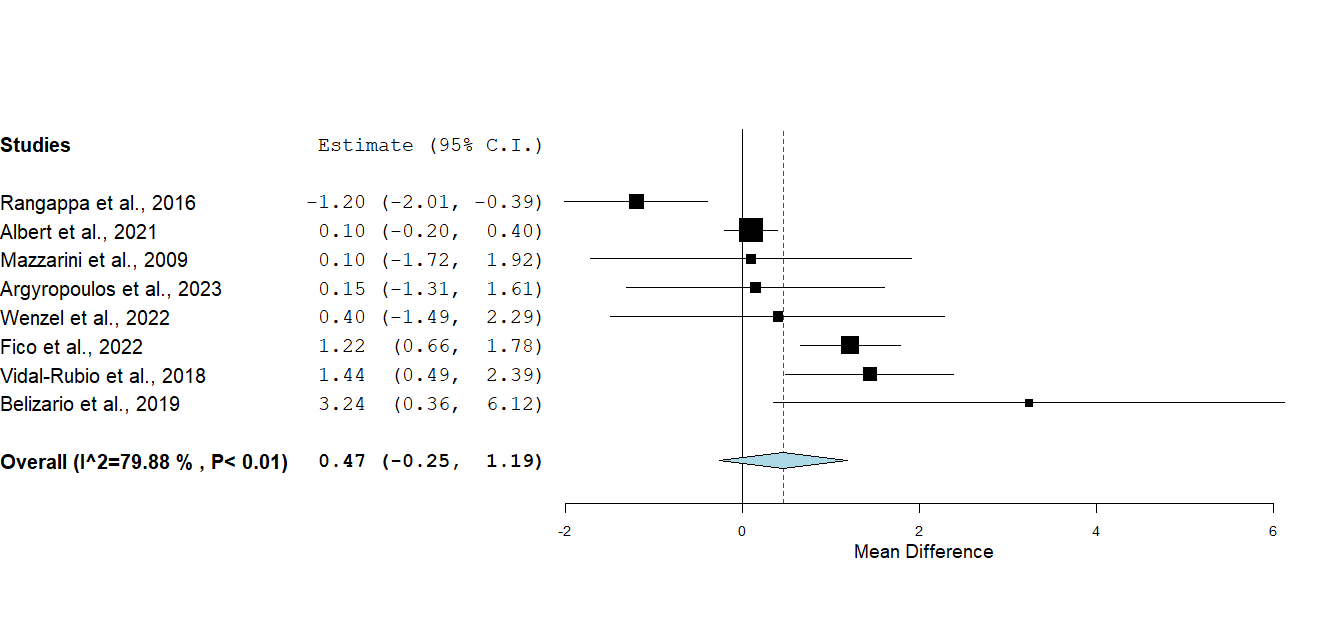


## **Supplementary Figure 20.** Mean difference in number of suicide attempts between participants with a hypomanic/manic predominant polarity and those with a depressive predominant polarity.


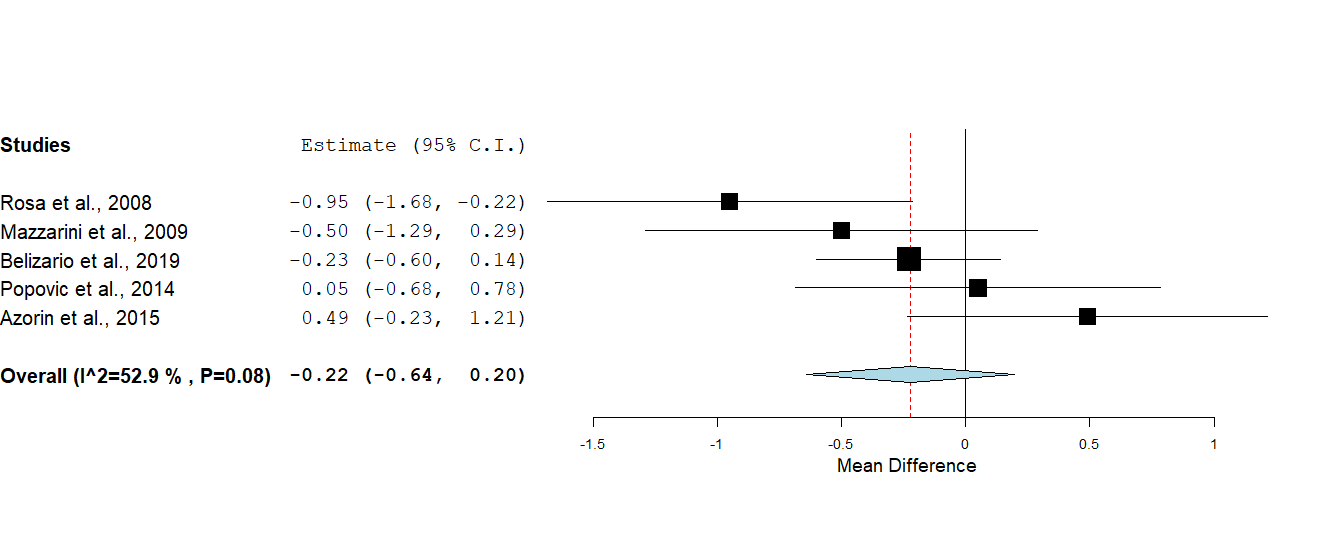


## **Supplementary Figure 21.** Alcohol use disorder in participants with a hypomanic/manic predominant polarity vs. those with a depressive predominant polarity.


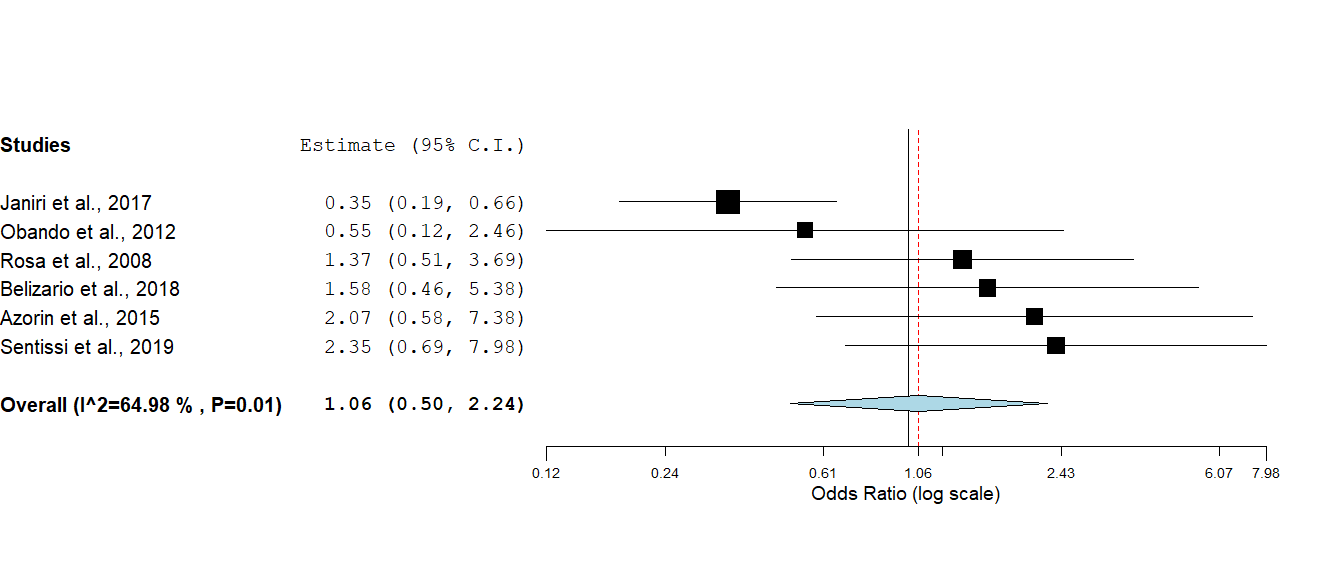


## **Supplementary Figure 22.** Substance use disorder in participants with a hypomanic/manic predominant polarity vs. those with a depressive predominant polarity.


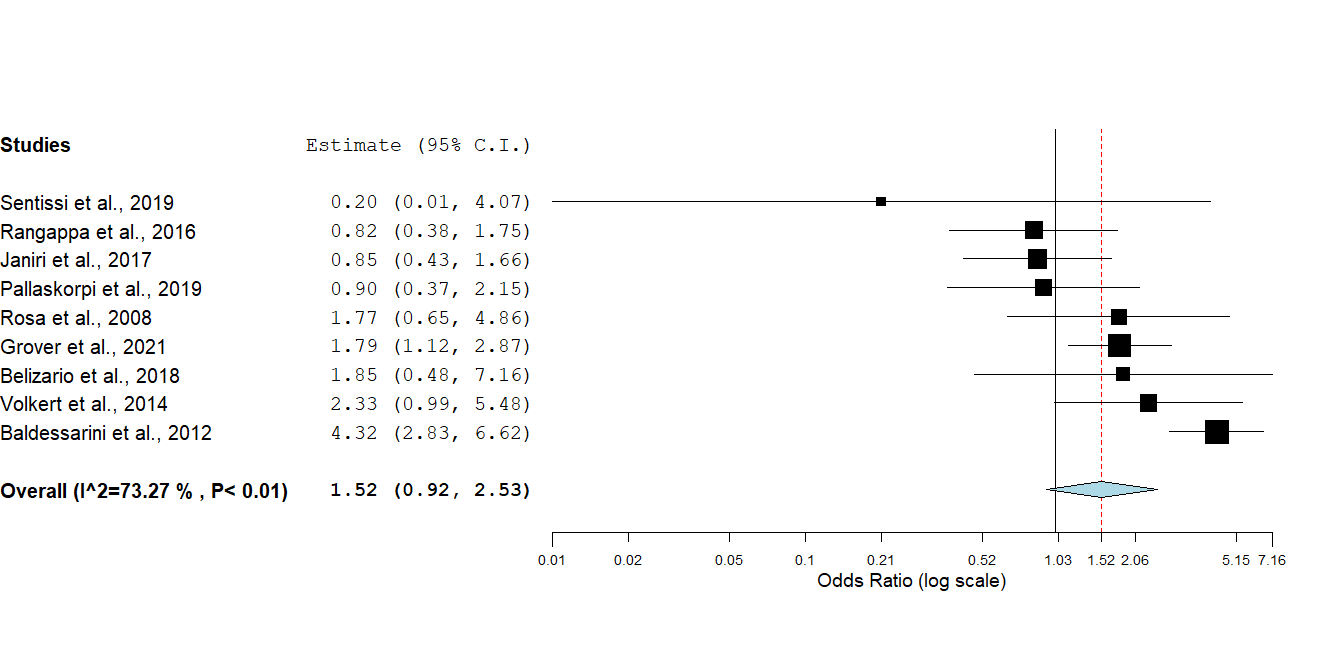


## **Supplementary Figure 23.** Family history of bipolar disorder in participants with a hypomanic/manic predominant polarity vs. those with a depressive predominant polarity.


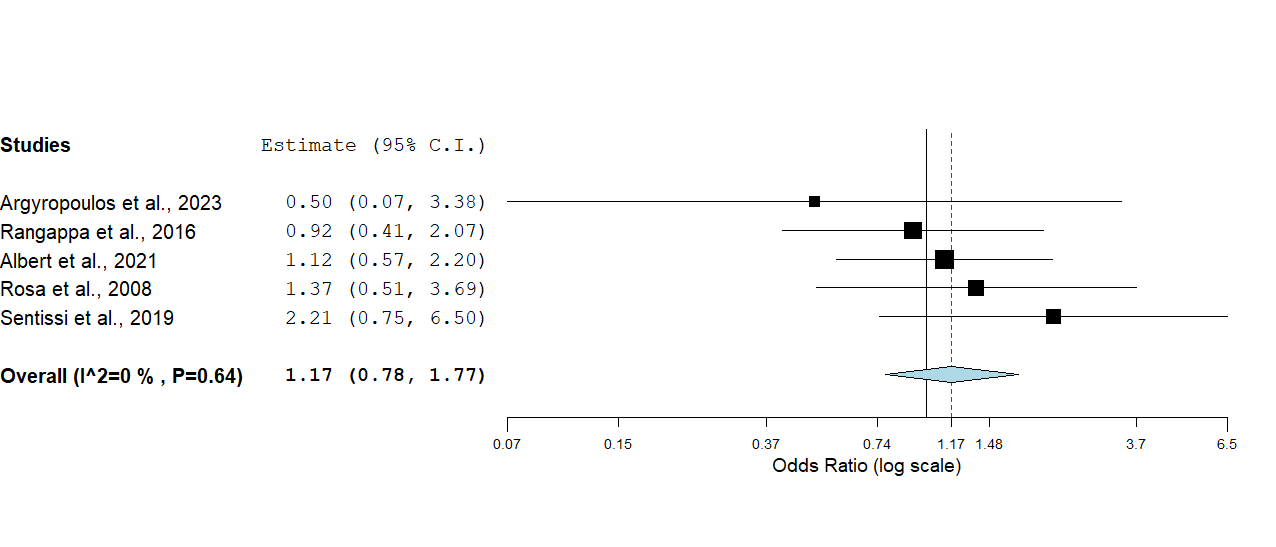


## **Supplementary Figure 24.** Family history of affective disorders in participants with a hypomanic/manic predominant polarity vs. those with a depressive predominant polarity.


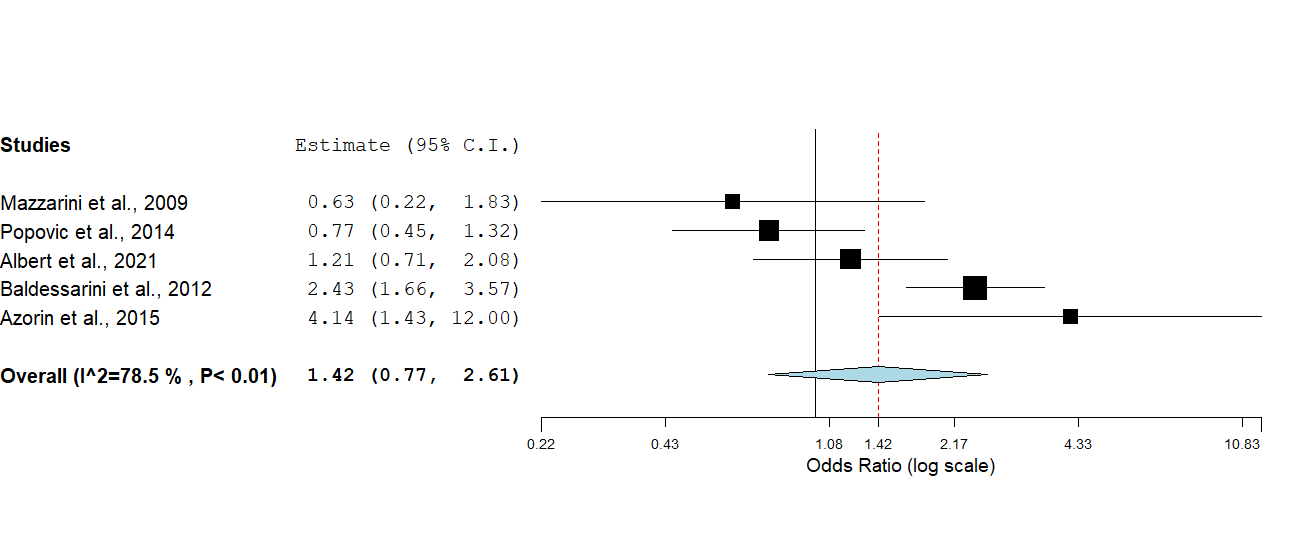


## **Supplementary Figure 25.** Family history of suicide in participants with a hypomanic/manic predominant polarity vs. those with a depressive predominant polarity.


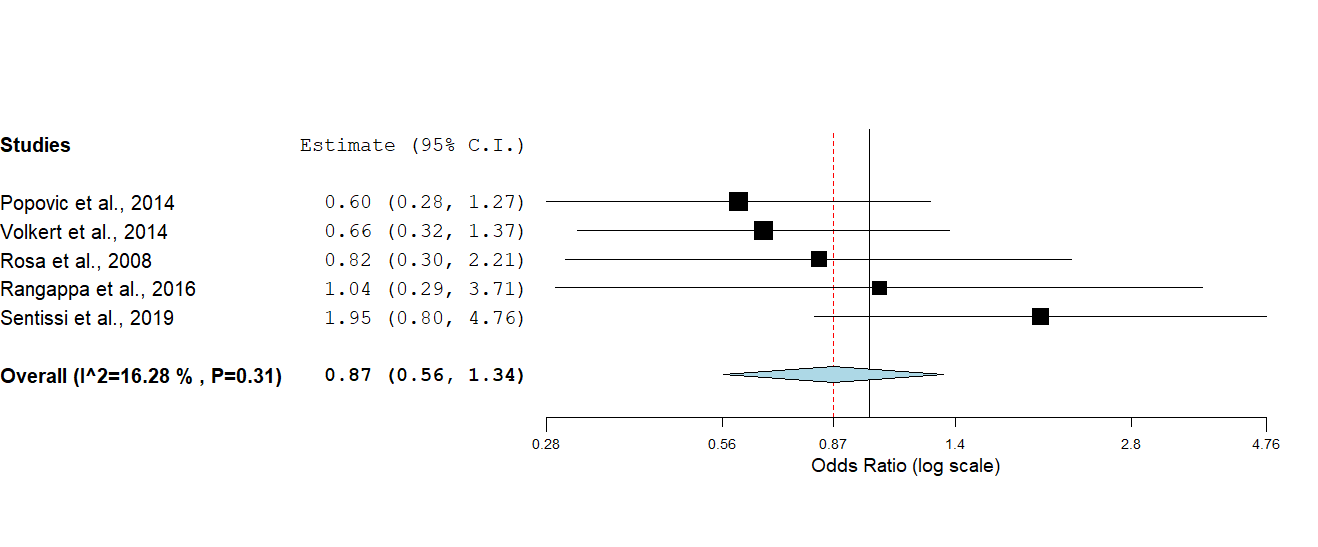

Supplement: Bartoli et al. supplementary material 1 — Bartoli et al. supplementary material [file S2056472424000516sup001.docx]
