# Supplementary material for: Prevalence and correlates of manic/hypomanic and depressive predominant polarity in bipolar disorder: systematic review and meta-analysis
Source: BJPsych Open. 2024 May 6;10(3):e100. doi: 10.1192/bjo.2024.51 (PMC11094450; doi:10.1192/bjo.2024.51)
Supplement: Bartoli et al. supplementary material 2 — Bartoli et al. supplementary material [file S2056472424000516sup002.docx]

**Supplementary Table 1.** Factors not associated with any predominant polarity: summary of findings.

| **Variable** | **k** | **N** | **Effect size ^§^**  **[95%CI]** | **p-value** | ***I^2^*** |
| --- | --- | --- | --- | --- | --- |
| Years of education | 6 | 820 | WMD = 0.01 years  [–0.73 to 0.76 years] | 0.97 | 21.0% |
| Unemployment | 5 | 829 | OR = 1.09  [0.79 to 1.49] | 0.61 | 0% |
| Duration of illness | 12 | 1,681 | WMD = –1.59 years  [–3.69 to 0.51 years] | 0.14 | 67.0% |
| Mixed polarity of first episode | 5 | 657 | OR = 1.20  [0.31 to 4.64] | 0.79 | 62.4% |
| Rapid cycling | 8 | 1,711 | OR = –0.66  [0.38 to 1.14] | 0.13 | 54.0% |
| Number of hospital admissions | 8 | 1,201 | WMD = 0.47 admissions  [–0.25 to 1.19 admissions] | 0.20 | 79.9% |
| Number of suicide attempts | 5 | 621 | WMD = –0.22 attempts  [–0.64 to 0.20 attempts] | 0.30 | 52.9% |
| Alcohol use disorder | 6 | 635 | OR = 1.06  [0.50 to 2.24] | 0.88 | 65.0% |
| Substance use disorder | 9 | 2,013 | OR = 1.52  [0.92 to 2.53] | 0.10 | 73.3% |
| Family history of bipolar disorder | 5 | 792 | OR = 1.17  [0.78 to 1.77] | 0.45 | 0% |
| Family history of any affective disorder | 5 | 1,221 | OR = 1.42  [0.77 to 2.61] | 0.26 | 78.5% |
| Family history of suicide | 5 | 908 | OR = 0.87  [0.56 to 1.34] | 0.52 | 16.3% |

**^§^** Hypomanic/manic predominant polarity vs. depressive predominant polarity

k = number of included studies; N = number of study participants; 95%CI = 95% confidence interval; OR = odds ratio; WMD = weighted mean difference.
